# Outcomes and complications reported from a multiuser canine hip replacement registry over a 10‐year period

**DOI:** 10.1111/vsu.13885

**Published:** 2022-09-05

**Authors:** Sumaya Allaith, Lydia J. Tucker, John F. Innes, Gareth Arthurs, Aldo Vezzoni, Shane Morrison, Jeremy Onyett, Christoph K. Stork, Philip Witte, Hamish Denny, Rob Pettitt, Andy P. Moores, Thomas Maddox, Eithne J. Comerford

**Affiliations:** ^1^ Department of Musculoskeletal and Ageing Sciences, Institute of Life Course and Medical Sciences University of Liverpool Liverpool UK; ^2^ ChesterGates Veterinary Specialists, Units E & F Cheshire UK; ^3^ Arthurs Orthopaedics, Towcester Veterinary Centre Towcester UK; ^4^ Clinica Veterinaria Vezzoni srl Cremona Italy; ^5^ Christchurch Veterinary Surgery Ipswich UK; ^6^ Abington Park Referrals, The Holcot Centre Northampton UK; ^7^ Orthovet Kent UK; ^8^ Cornwall Veterinary Referrals, Penmellyn Veterinary Group Cornwall UK; ^9^ Denny Veterinary Orthopaedic Referrals, Cedar House Bristol UK; ^10^ Small Animal Teaching Hospital, Leahurst Campus, School of Veterinary Science University of Liverpool Neston UK; ^11^ Anderson Moores Veterinary Specialists, Bunstead Barns, Poles Lane, Hursley Hampshire UK

## Abstract

**Objective:**

To report outcomes and complications associated with total hip replacements (THR) using a multiuser canine hip registry (CHR) and owner‐administered questionnaire.

**Study design:**

Prospective longitudinal clinical study.

**Animals:**

Dogs (*n* = 1852).

**Methods:**

Total hip replacement cases submitted to a CHR were reviewed. An online questionnaire including an adapted “Liverpool Osteoarthritis in Dogs” (LOAD) score was e‐mailed to owners. Data were analyzed to determine associations between clinical variables and the agreement by veterinary surgeons and owners for complications.

**Results:**

A group of 1329 (72%) dogs had unilateral THRs and another group of 523 (28%) dogs had bilateral THRs, resulting in 2375 THRs. Indications included hip dysplasia and osteoarthritis (*n* = 2028/2375, 85%). Implants were manufactured by Kyon (*n =* 1087, 46%), BioMedtrix CFX (*n* = 514, 22%), BioMedtrix hybrid (*n* = 264, 11%), BioMedtrix BFX (*n* = 221, 9%), and Helica (*n* = 107, 4.5%). Median veterinary surgeon and owner follow up were 1328 and 900 days respectively. Postoperative LOAD scores (21 ± 9) reported by 461 owners improved compared to preoperative scores (11 ± 9) (*P* < .001). Veterinary surgeons reported complications in 201/2375 (8.5%) THRs and owners in 107/461 (23%) THRs, with moderate agreement (weighted kappa = 0.44). No associations were identified between complications and weight, age, sex, or breed. BioMedtrix BFX and Helica implants were associated with increased complications (*P* = .031) when used for revisions of femoral head and neck excisions.

**Conclusion:**

Excellent outcomes, including improved canine mobility, were reported after THRs. Complications were underreported by veterinary surgeons compared to owners in this first multiuser CHR.

**Clinical significance:**

Canine THRs are safe, effective procedures but THR implants should be carefully selected when revising femoral head and neck excisions.

## 1 INTRODUCTION

Total hip arthroplasty/replacement (THR) is a surgical option for treating coxofemoral (hip) joint conditions such as hip dysplasia (HD) and osteoarthritis (OA).^1^ A decision to perform THR largely depends on the severity of clinical hip disease, a failure of conservative management, veterinary surgeon advice, and the client's wishes and ability to cover the costs.^2^

Complications following canine orthopedic procedures, such as THR, have been categorized as minor, major, or catastrophic.^3^ Commonly reported minor complications include wound dehiscence, temporary mild sciatic neuropathy, femoral medullary infarction, and major complications include femoral fracture, patellar luxation, acetabular cup displacement, severe sciatic neuropathy, septic and aseptic loosening.^4^, ^5^, ^6^, ^7^, ^8^ Cemented implants (eg, BioMedtrix CFX (Cemented fixation), Whippany, New Jersey, USA) have the femoral and acetabular implants cemented producing a bone‐cement‐implant interface, whereas cementless implants (eg, BioMedtrix BFX (Biologic fixation), Whippany, New Jersey, USA) are press fit without a cohesive agent such as cement allowing bone ingrowth onto and into the prosthesis at the bone‐implant interface.^1^, ^9^, ^10^ The Zurich Cementless system (Kyon AG, Zurich, Switzerland), has been successfully used in dogs and has a locking screw technology to avoid micromotion of the femoral component which, with the cup, has a hydroxyapatite coating allowing bone ingrowth into and onto the protheses as described above.^11^, ^12^

More recently, the use of the Helica (Innoplant Veterinary, Hannover, Germany) implant system has been reported in canine THR, most commonly consisting of a cementless acetabular cup and a cemented femoral implant.^13^ These combinations of both cemented and cementless implants in the same THR surgical procedure (hybrid THR) have further been reported.^14^ Previous studies^15^, ^16^, ^17^, ^18^, ^19^ reported both the success and complication rates of each THR implants individually, however, case numbers in these studies are relatively small, making it challenging to draw firm conclusions on the association between different implants and complications.^17^, ^21^

Several databases have been developed to monitor and report joint arthroplasty procedures in human surgery.^22^ The reliability of different total hip arthroplasty implants has been previously reported using cemented and cementless implants.^23^ It has been shown in human patients that the average lifespan for a hip implant is around 10‐15 years.^24^ Due to the extended timeframe, there is thus a duty of care and responsibility upon clinicians to monitor both performance and quality of implants.^24^

The British Veterinary Orthopedic Association – University of Liverpool (BVOA‐UoL) canine hip registry (CHR), officially launched in January 2010,^15^ is an online database that aims to collect information from multiple veterinary THR surgeons, on canine (and, more recently, feline) THRs including the animal's medical history, implants used, and surgical complications.^15^ Previous studies analyzed variables derived from the BVOA‐UoL CHR between January 2010 and December 2012, assessing outcomes with an annual online owner‐administered questionnaire, including the client reported outcomes measure (Liverpool Osteoarthritis in Dogs, LOAD).^15^, ^20^, ^25^ However, in the context of THR, the follow up in these studies was relatively short (2‐3 years).^15^, ^20^ Most other published studies to date on canine THR have also only reported cases from a single center.^26^, ^27^, ^28^, ^29^

The aim of this study was therefore to assess prognostic factors, outcome, and complications from the BVOA‐UoL CHR database and the owners of registered dogs over a 10‐year period. We planned to do this by (1) reporting dog variables (age, body weight, breed, sex, surgical indications and implant type), from the BVOA‐UoL CHR database, (2) identifying associations between owner‐reported complications and dog variables, and (3) by analyzing the overall outcome and improvement in mobility before and after THR from online owner‐administered questionnaire of registered dogs on the BVOA‐UoL CHR database.

## 2 MATERIALS AND METHODS

### 2.1 Case selection

The BVOA‐UoL CHR was granted full institutional ethical approval (VREC856). A material transfer agreement (MTA) was signed between the BVOA‐UoL CHR database host institution and participating veterinary surgeons (VS) to ensure protection of their data and participation. Fully informed VS (performing the THRs) and owner consent was obtained before cases could be registered on the CHR database. Further detail on establishment of the BVOA‐UoL CHR has been reported previously.^15^, ^20^ Data on each THR were initially submitted to a virtual online collaboration and learning (VOCAL) site (January 2010‐December 2018) and then transferred to a dedicated Microsoft SharePoint site (December 2018‐June 2020) which external users could access on invitation. The CHR database included VS and owner's contact information (veterinary practice name, owner's email address, telephone contact number, surname, initials, home address) and patient details (dog's name, age, sex, breed, THR date, indication for surgery, complications, date of complication, actions taken for complications, death records, and a LOAD score to assess the dog's mobility before THR).^25^ Other information included implants for surgery, implant size, and cementing techniques. The CHR database was exported in June 2020 to Excel (Microsoft Excel 2016, Microsoft, Redmond, Washington) and the data were reviewed. Participating VS voluntarily added complication reports after creating the initial case record. Complications were categorized into minor, major, or catastrophic depending on the severity of the complication according to the definitions previously reported.^3^

### 2.2 Online owner‐administered questionnaire following THR

An electronic survey was sent to 1568 out of 1852 owners via joint information systems committee (JISC) to their e‐mail addresses in November 2019 (https://uol.csd/software-support/survey-software/). Both an English version and a translated version of the electronic survey were sent to owners. Owners who did not have a valid e‐mail address or who had informed us via email that their dog had died were not contacted (*n =* 284). The cause of death was reported by pet owners via e‐mail. Clients who did not respond to the first survey were sent a second reminder in January 2020. The JISC software data were afterwards exported into Excel spreadsheet to be analyzed in May 2020 (Microsoft Excel 2016, Microsoft). The questionnaire was based on previous studies and was divided into 4 sections.^15^, ^20^ Part 1 included owner details (date, owner's surname, and a timeframe for THR). Part 2 consisted of patient information (dog's name, age, breed, sex, and which hip joint underwent THR). Part 3 had 15 questions asking about the dog's status before and after THR (modified from the LOAD question format onto an electronic survey),^25^ which included the timeline for mobility problems, diseases diagnosed other than hip disease, medications received, the dog's activity, and willingness to exercise before THR, complications that occurred after THR and their treatment, and overall owner satisfaction. Part 4 consisted of 13 questions to assess the LOAD score after THR (modified from the LOAD question format onto an electronic survey).^25^

### 2.3 Inclusion and exclusion criteria

The inclusion criteria for this study were (1) registration of the dog and owner details, as well as indications for THR and implants used, with full owner and VS consent onto the CHR database and (2) completion of an online owner‐administered questionnaire by owners of registered dogs on the CHR database. Owners whose dogs were recorded as dead or who had an incorrectly registered e‐mail address were excluded from receiving an online owner‐administered questionnaire.

### 2.4 Descriptive and statistical analysis

Data were obtained from the online owner‐administered questionnaire and BVOA‐UoL CHR. GraphPad Prism statistical software (GraphPad Prism 9.0., San Diego, California) was used to obtain descriptive statistics such as mean, standard deviation, and median value for the dog's age, body weight, preoperative LOAD score, and postoperative LOAD score. Mean and standard deviation are displayed as (mean ± SD). Data were tested for normality using the Kolmogorov‐Smirnov test. Statistical analysis was performed to evaluate differences between the preoperative and postoperative LOAD scores. The analysis involved comparing 2 groups (LOAD score pre THR vs. LOAD score post THR) at 12 months intervals. Statistical analysis was performed to compare the preoperative LOAD score and postoperative LOAD score using a paired nonparametric *t*‐test (Wilcoxon matched‐pairs signed rank test) as the data were not normally distributed. Statistical significance was set at *P* < .05.

SPSS statistical software (IBM SPSS Statistics 26, Armonk, New York) was used to identify associations between owner and VS reported complications and dog variables (age, bodyweight, breed, sex, surgical indications and implant type). The agreement was assessed between the incidence of complications reported by the owner and the VS with Cohen's kappa (k value) coefficient, followed by a weighted kappa (k value) to assess agreement between the severity of complications reported by the owner and the VS. Associations between each independent variable and the incidence of complications were assessed using logistic regression analysis. Univariable binomial logistic regression was used to calculate measures of strength of association (ORs and 95% CI) for each variable with the presence of a complication reported by the owner or/and the VS. Variables showing some evidence of association (*P* < .2) on univariable analysis were included in a multivariable binomial regression for the same outcome, constructed in a backwards stepwise fashion with variables retained if *P* < .05. Statistical significance was set at *P* < .05 (IBM SPSS Statistics 26). Data for these analyses were obtained from both the online owner‐administered questionnaire and BVOA‐UoL CHR.

## 3 RESULTS

### 3.1 BVOA‐UoL CHR database

#### 3.1.1 Demographics

**FIGURE 1:**
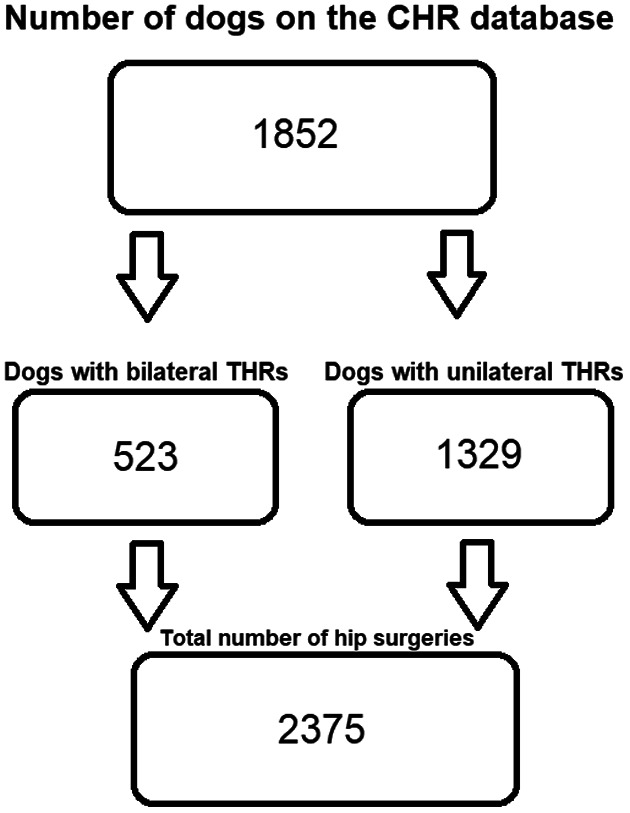
A flowchart representing the number of THR surgeries registered on the CHR database. THR, total hip replacement; CHR, canine hip registry

Thirty veterinary practices submitted THR cases to the BVOA‐UoL CHR. The submission rates from individual clinics are shown in Supplementary material 1. The CHR database consisted of 1852 dogs in June 2020. There were 1329 (72%) dogs that had unilateral THRs and 523 (28%) dogs that had bilateral THRs, resulting in 2375 hip replacement surgeries in total (Figure 1). The number of dogs' deaths recorded over the 10‐year period was 185/1852 (10%). Eleven dogs out of 185 were euthanatized due to complications of the THR (6%). The remaining dogs (*n =* 174/185, 94%) were reported to have died from causes not related to the THR (Supplementary material 2).

**FIGURE 2:**
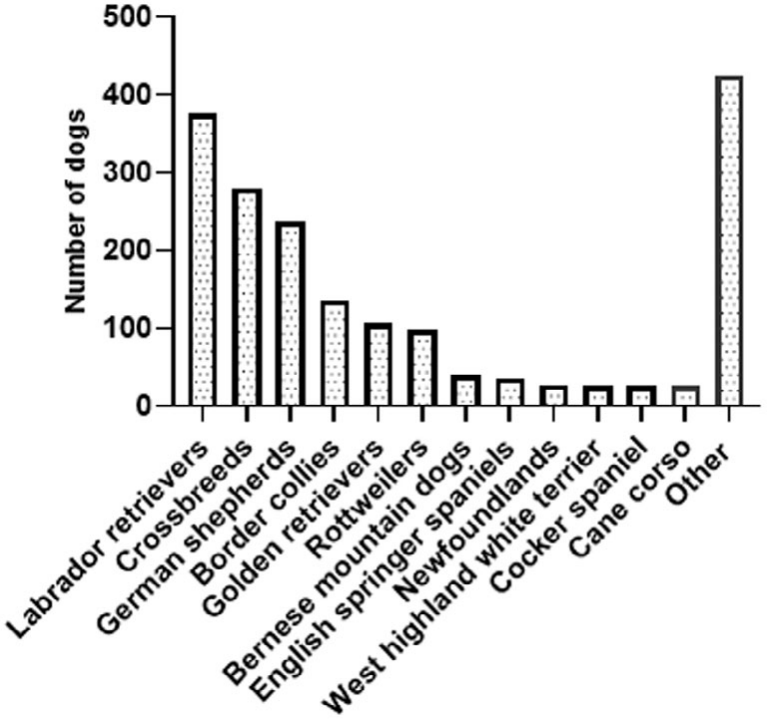
The most commonly presented dog breeds on the CHR database that have had THRs

Registered CHR dogs had an average age of 43.2 months ±34.8, median 24 months, and weighed 29.5 ± 10.5, median 29 kilograms. Out of 1852 dogs, there were 999 male dogs, intact (*n =* 553, 30%) and neutered (*n =* 446, 24%) and 831 female dogs, intact (*n =* 336, 18%) and neutered (*n =* 495, 27%). Twenty‐two dogs (1%) did not have their sex specified. The breeds recorded were Labrador retrievers (*n =* 376/1852, 20%), crossbreeds (*n =* 280/1852, 15%), German shepherds (*n =* 238/1852, 13%), Border collies (*n =* 136/1852, 7%), golden retrievers (*n =* 106/1852, 6%), Rottweilers (*n =* 99/1852, 5%), Bernese mountain dogs (*n =* 41/1852, 2%), English springer spaniels (*n =* 36/1852, 2%), Newfoundlands (*n =* 28/1852, 2%), West Highland white terriers (*n =* 26/1852, 1%), cocker spaniels (*n =* 26/1852, 1%), and cane corso (*n =* 26/1852, 1%) (Figure 2). Other dog breeds (*n =* 425/1852, 23%) are detailed in supplementary material 3. The breed was not recorded for 9 dogs in the database.

#### 3.1.2 Surgical implants

**FIGURE 3:**
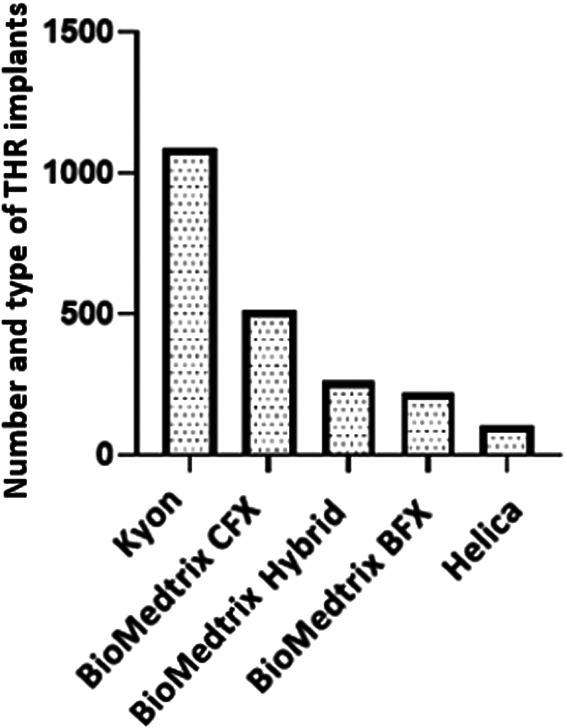
The numbers of implants from different THR systems present on the CHR database. THR, total hip replacement, CFX, Cemented fixation, BFX, Biologic fixation.

**FIGURE 4:**
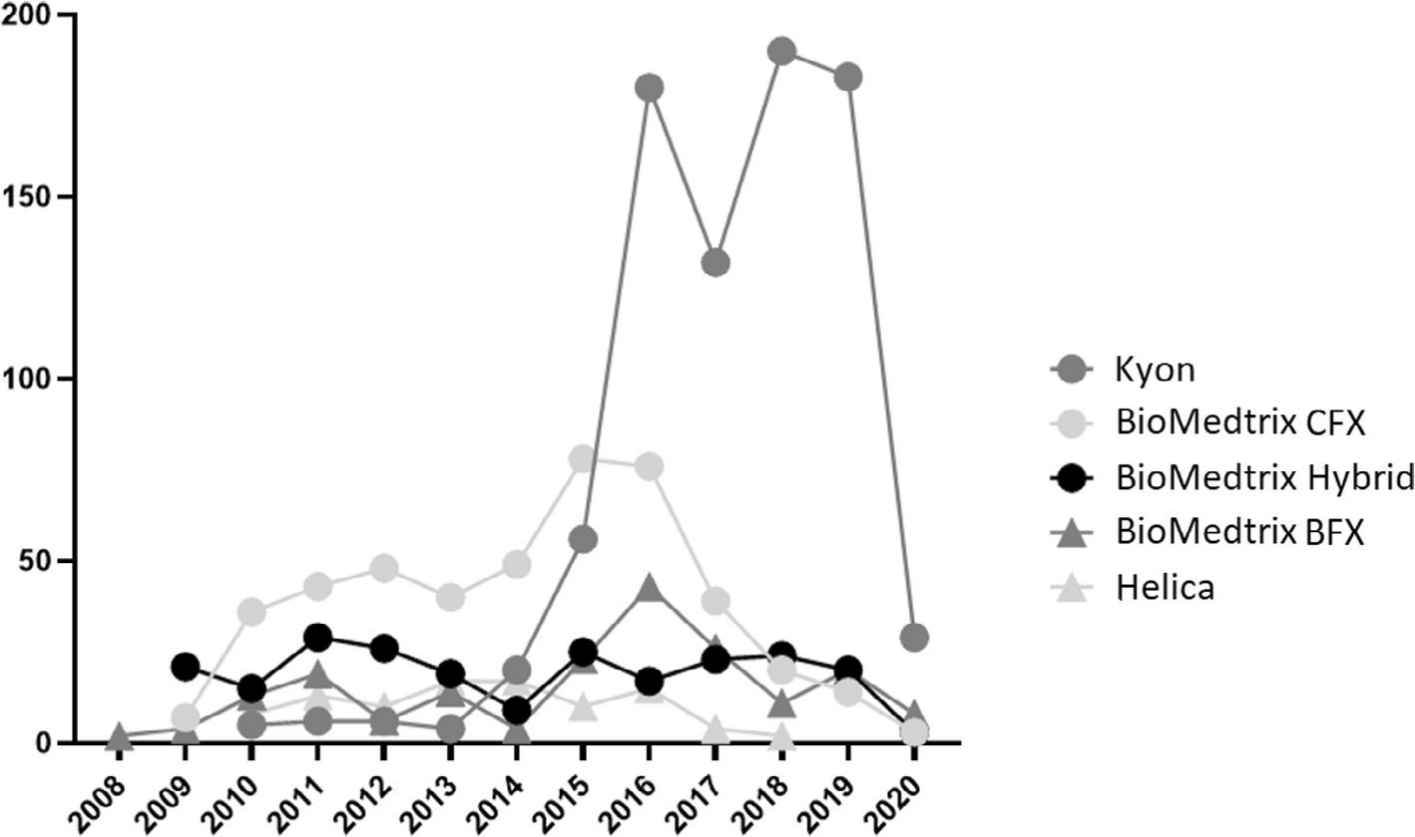
The increase in the number of dogs over the years in relation to the implant type on the CHR database. CFX, Cemented fixation, BFX, Biologic fixation.

Implant systems reported were Kyon (*n =* 1087/2375, 46%), BioMedtrix CFX (*n =* 514/2375, 22%), BioMedtrix Hybrid (*n =* 264/2375, 11%), BioMedtrix BFX (*n =* 221/2375, 9%), and Helica (*n =* 107/2375, 4.5%). (Figure 3). One hundred and eighty‐two (182/2375, 8%) of the implants were not specified. The number of centers submitting data for each prosthesis type is shown in Table 1. BioMedtrix Modular CFX Micro & Nano Hip implants were used in 142/2375 hips (6%). Implants and their sizes are listed in Supplementary material 4. Figure 4 demonstrates the implant types and systems registered over the 10‐year period.

**TABLE 1:** The number of veterinary practices submitting data for each canine total hip replacement implant type. CFX, Cemented fixation, BFX, Biologic fixation.

Prosthesis type	Number of centers
Kyon	3
BioMedtrix CFX	17
BioMedtrix BFX	8
BioMedtrix Hybrid	13
Helica	3

#### 3.1.3 Surgical indications

**FIGURE 5:**
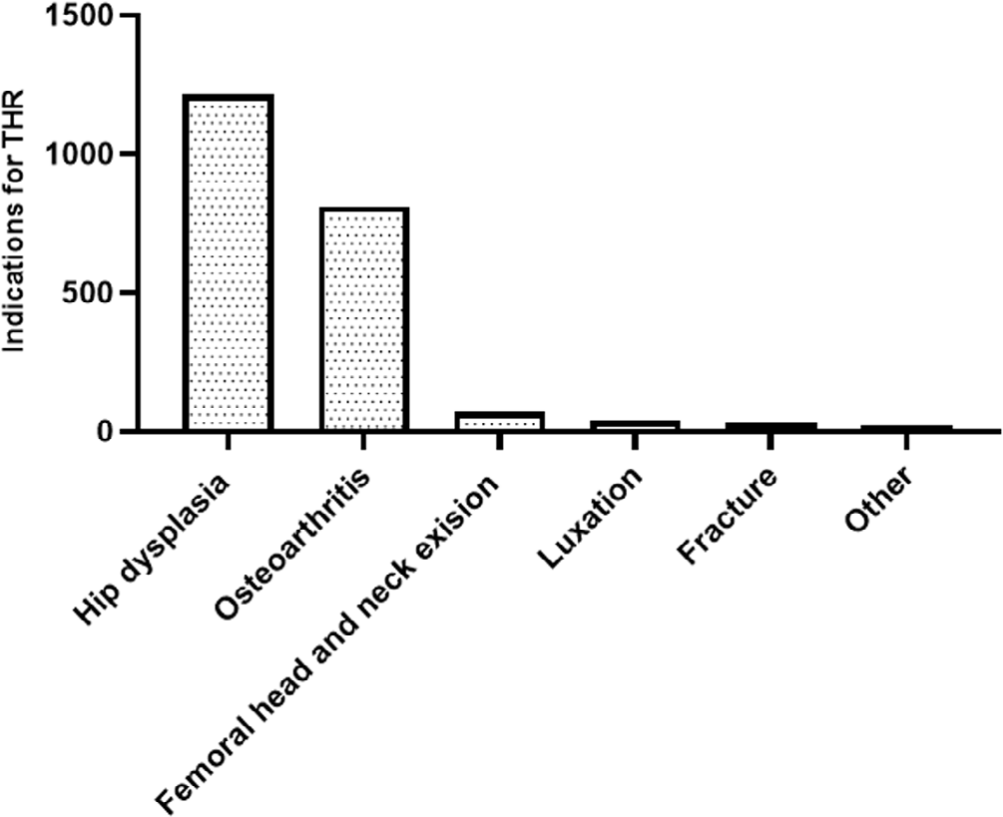
Indications for THR on the CHR database. THR, total hip replacement.

Indications included hip dysplasia (*n =* 1216/2375, 51%), osteoarthritis (n = 812/2375, 34%), femoral head and neck excision (*n =* 71/2375, 3%), coxofemoral luxation (*n =* 40/2375, 2%), fracture (*n =* 33/2375, 1%), and other surgical indications (*n =* 19/2375, 1%) (Figure 5). One hundred and eighty‐four (184/2375, 8%) indications were not specified.

#### 3.1.4 Complications – Veterinary surgeon‐reported

**FIGURE 6:**
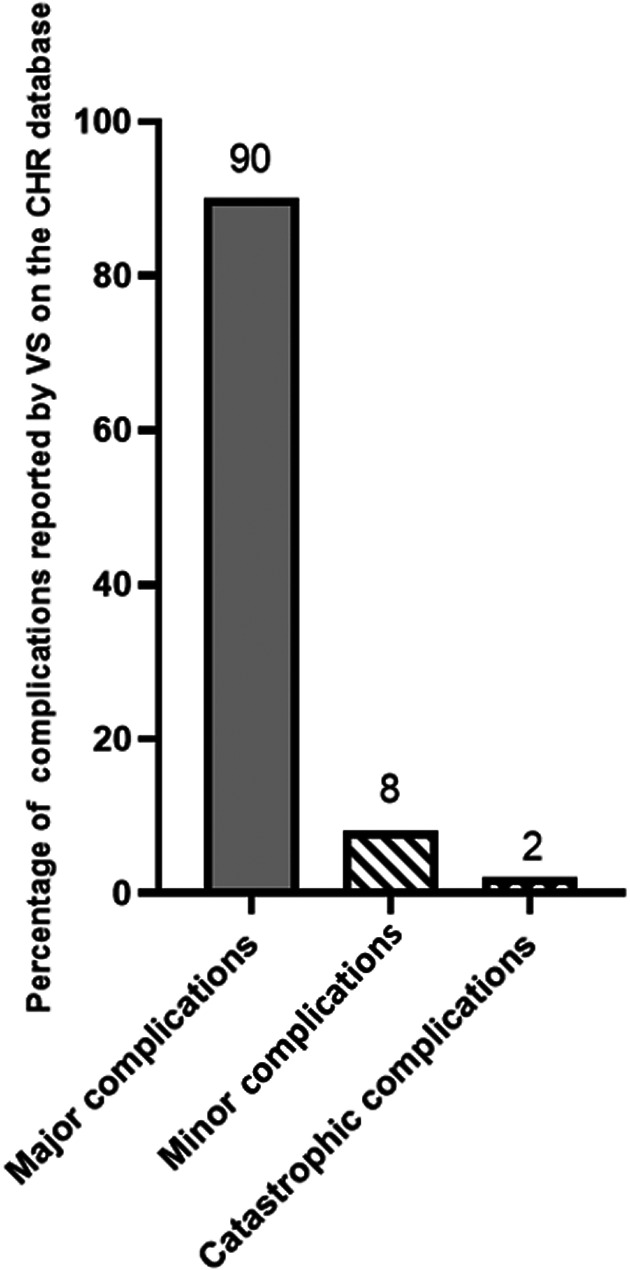
Percentage of complications reported by veterinary surgeon on the CHR database. Complications were categorized according to severity (major/minor/catastrophic). CHR, canine hip registry. VS, veterinary surgeon

**FIGURE 7:**
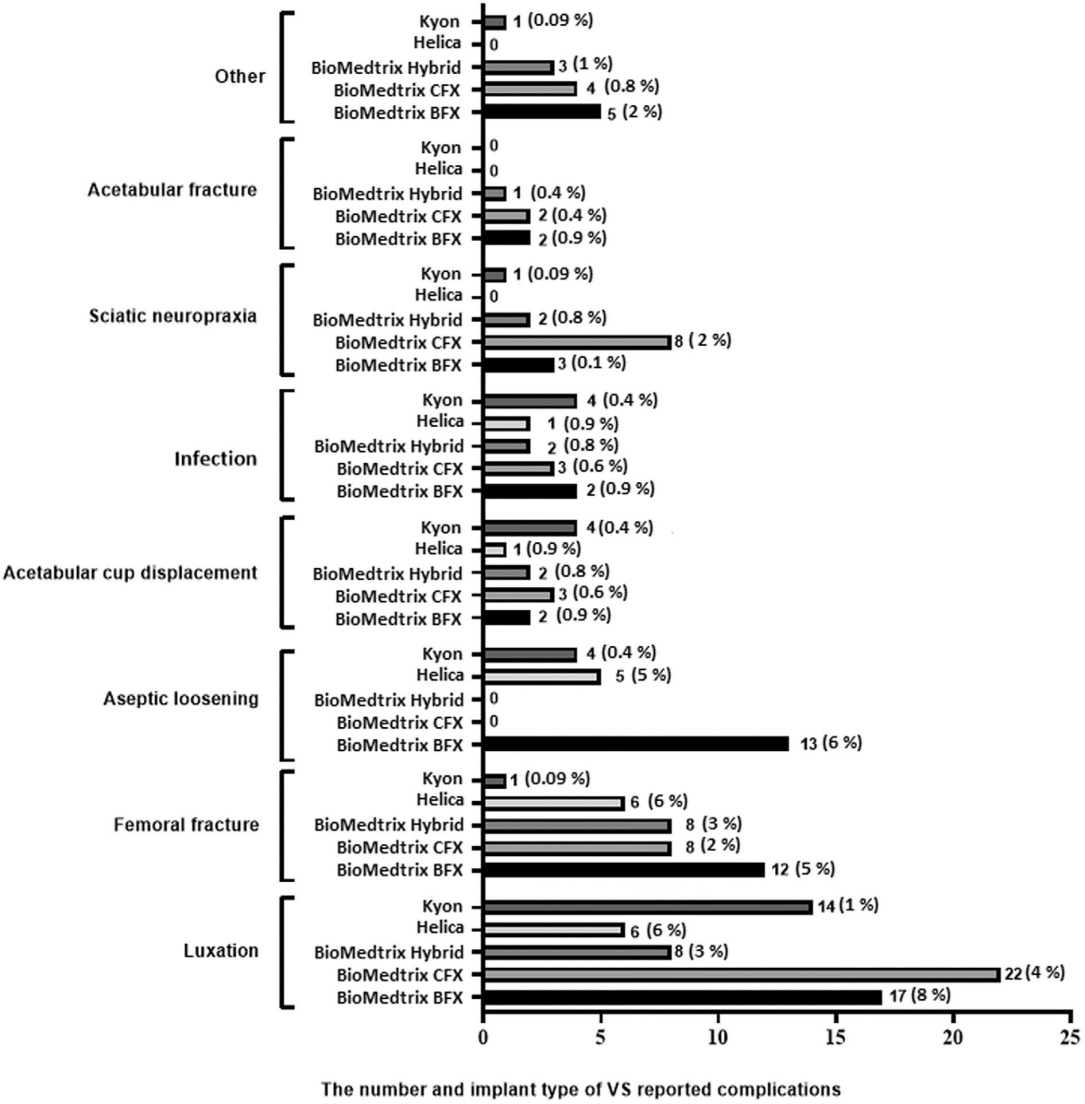
Veterinary surgeon reported complications related to different THR implant systems. THR, total hip replacement. VS, veterinary surgeon, CFX, Cemented fixation, BFX, Biologic fixation.

The median follow up by VS of THRs on the CHR database was 1328 days (range 203‐3932). The VS reported complication rate was 8.5% (*n =* 201/2375). Complications consisted of 1 complication per THR (*n =* 182), 2 complications per THR (*n =* 18), or 3 complications per THR (*n =* 1), consisting of 221 minor and major complications in total. Complications and their classifications are categorized in Figure 6. Minor complications included sciatic neuropraxia (*n =* 15/221, 7%), neuropathic pain (*n =* 1/221, 0.5%), and synoviocoele (*n =* 1/221, 0.5%). Major complications included luxation (*n =* 80/221, 36%), fracture (*n =* 39/221, 18%), aseptic loosening (*n =* 29/221, 13%), femoral acetabular cup loosening (*n =* 23/221, 10%), infection (*n =* 16/221, 7%), acetabular fracture (*n =* 5/221, 2%), implant failure (*n =* 3/221, 1%), wound dehiscence (*n =* 1/221, 0. 5%), osteosarcoma (*n =* 1/221, 0.5%), extraosseous cement granuloma (*n =* 1/221, 0.5%), implant displacement (*n =* 1/221, 0.5%), and poor function of implant (*n =* 1/221, 0.5%). Actions taken for minor and major complications are reported in supplementary material 5. Four out of 221 (2%) complications were classified as catastrophic complications. One dog died from intraoperative cardiac arrest (*n =* 1/221, 0.5%), 3 other dogs were euthanatized due to femoral fracture (*n =* 2/221, 1%) and aseptic loosening (*n =* 1/221, 0.5%) respectively. Surgical implants associated with complications per hip were BioMedtrix CFX (*n =* 49/201, 24%), BioMedtrix BFX (*n =* 42/201, 21%), Hybrid BioMedtrix (*n =* 24/201, 12%), Kyon (*n =* 32/201, 16%), and Helica (*n =* 22/201, 11%). In 32/201 (16%) reported complications, the surgical implants were not recorded. Complications and their association with surgical implants are illustrated in Figure 7.

### 3.2 Online owner‐administered questionnaire

**FIGURE 8:**
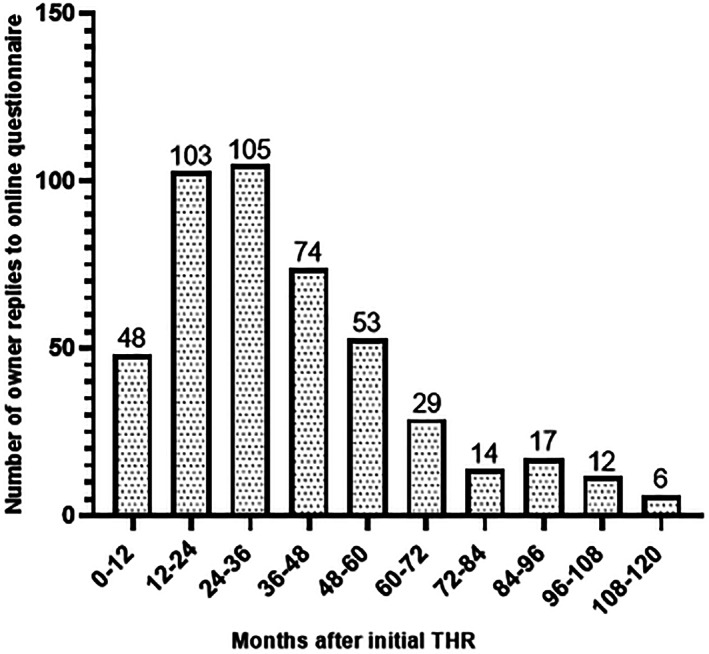
Length of time owners of dogs registered on the CHR database replied to the online annual questionnaire following THR. CHR, canine hip registry; THR, total hip replacement

*Response rate, and time of survey completion post THR*: The response rate of the online owner‐based assessment questionnaire was 29% (461/1568). The timeline of when owners completed this survey after the initial operation are detailed in Figure 8. The median follow up by owners was 900 days (range 180‐3600). *Number of THRs, age and weight of dogs*: THRs were performed on either only the left hip (*n =* 144/461, 31%), or the right hip joint (*n =* 130/461, 28%) or bilaterally (*n =* 187/461, 41%). At the first THR, age was 35 ± 29 months, median 24 months, with 56% of the dogs being less than or equal to 24 months whilst bodyweight was 26.7 kg ± 10.6 kg, median 27 kg. At the second THR, dogs had an age of 38 ± 31 months, median 24 months, and bodyweight of 29.3 kg ± 8.9 kg, median 28 kg. There were 206 females; 76/461 were intact (16%) and 130/461 were neutered (28%). There were 249 male dogs; 132/461 were intact (29%) and 117/461 were neutered (25%) males. 6/461 (1%) dogs did not have their sex recorded. The most frequently represented breed types were crossbreed (*n =* 88/461, 19%), Labrador retriever (*n =* 83/461, 18%), German shepherd (*n =* 56/461, 12%), Border collie (*n =* 44/461, 10%), golden retriever (*n =* 30, 6.5%), springer spaniel (*n =* 27, 6%), terrier (*n =* 26, 6%), Rottweiler (*n =* 10, 2%) and otterhound (*n =* 7, 1.5%). Two out of 461 (0.4%) dogs did not have a breed type recorded. Other less frequent breeds (*n =* 88/461, 19%) reported on the owner‐based assessment questionnaire are provided in Supplementary material 6. *General health data*: 92 owners (92/461, 20%) reported that their dogs were diagnosed with other problems in addition to their hip disease (Supplementary material 7). Some dogs (*n =* 24/461, 5%) only had nutraceuticals (with no analgesic properties) prior to THR. Less than half of the owners (220/461, 48%) reported that their dog had any medication prior to THR. Medications listed were NSAIDs only (*n =* 132/220, 60%), more than 1 medication (*n =* 58/220, 26%), analgesics only (*n =* 22/220, 10%), and other medications (*n =* 8/220, 4%). Prior to their THR, the dogs had previously been suffering from mobility problems for up to 6 months (*n =* 157/461, 34%), 6‐12 months (*n =* 137/461, 30%), 12‐24 months (*n =* 60/461, 13%), 24‐36 months (*n =* 18/461, 4%), and more than 36 months (*n =* 89/461, 19%).

**FIGURE 9:**
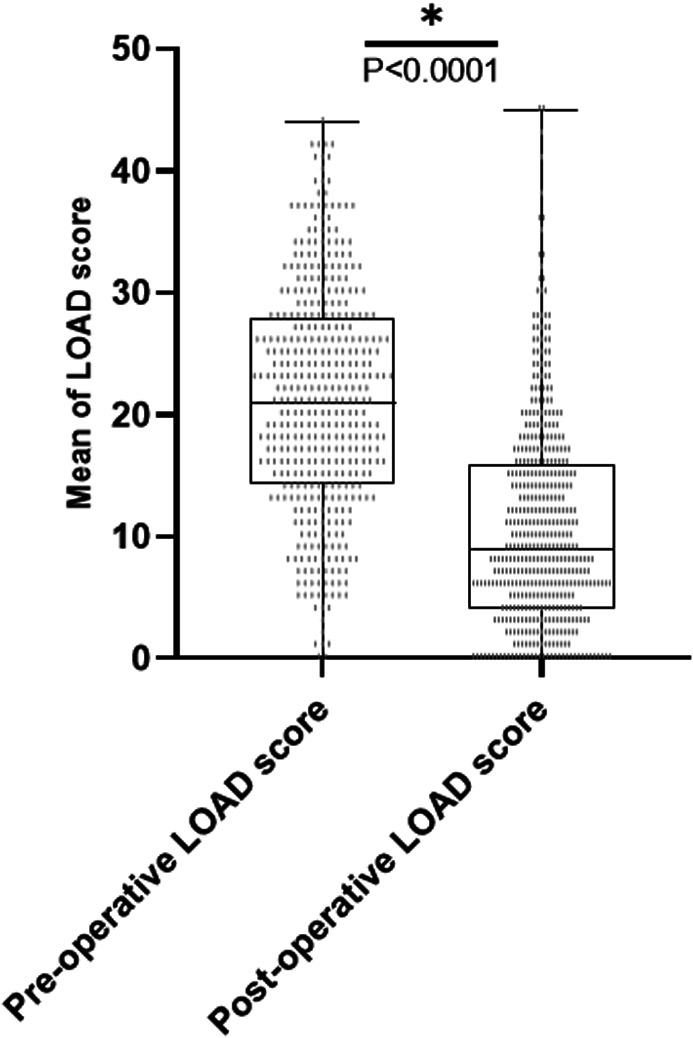
Minimum to maximum values of preoperative (*n =* 424) and postoperative (*n =* 458) LOAD scores. There is a significant difference between the preoperative and postoperative LOAD scores (*P* < .0001). Significance is set at *P* < .05. LOAD, Liverpool Osteoarthritis in Dogs

**FIGURE 10:**
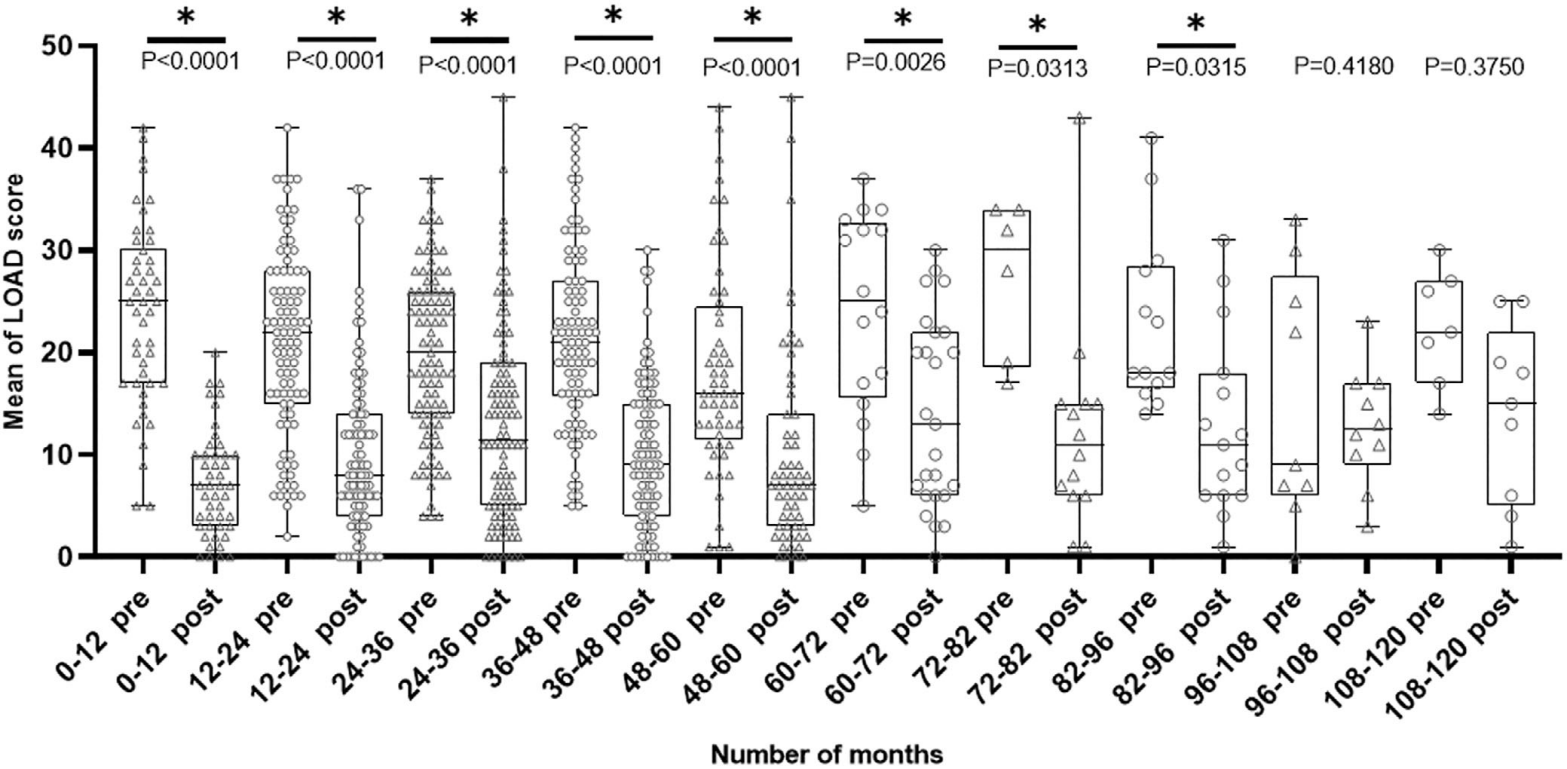
Minimum to maximum values of preoperative and postoperative LOAD scores within different time frames since the date of the initial THR. Significance is set at *P* < .05. Submission from owners include: pre 0‐12 (*n =* 47), post 0‐12 (*n =* 47), pre 12‐24 (*n =* 94), post 12‐24 (*n =* 93), pre 24‐36 (*n =* 92), post 24‐36 (*n =* 99), pre 36‐48 (*n =* 91), post 36‐48 (*n =* 92), pre 48‐60 (*n =* 54), post 48‐60 (*n =* 59), pre 60‐72 (*n =* 17), post 60‐72 (*n =* 26), pre 72‐82 (*n =* 7), post 72‐82 (*n =* 15), pre 82‐96 (*n =* 14), post 82‐96 (*n =* 16), pre 96‐108 (*n =* 10), post 96‐108 (*n =* 11), pre 108‐120 (*n =* 8), post 108‐120 (*n =* 10). LOAD, Liverpool Osteoarthritis in Dogs. Pre, preoperative LOAD score. Post, postoperative LOAD score. THR, total hip replacement

*Satisfaction outcome*: In 294/461 (64%) of cases, owners recorded their satisfaction with the outcome of THR as very good, 106/461 (24%) as good, 28/461 (6%) as fair, whereas a minority rated their satisfaction as poor (*n =* 14/46, 3%) and very poor (*n =* 16/461, 3%). *LOAD scores*: LOAD scores before THR (preoperative) were obtained from the BVOA‐UoL CHR, whereas LOAD scores after THR (postoperative) were obtained from the owner‐based assessment questionnaire. The mean LOAD score before THR was 21 ± 9 median 21, whereas the mean LOAD score after THR was 11 ± 9, median 9. There was a difference between LOAD scores before and after THR (*P* < .0001) (Figure 9). The LOAD scores for each timeline are illustrated in Figure 9. The LOAD scores at different timepoints after THR were decreased compared to LOAD scores before THR, although the LOAD score after THR tended to increase the longer the duration following surgery (Figure 10).

#### 3.2.1 Complications – Veterinary surgeon (VS) and owner‐reported

**FIGURE 11:**
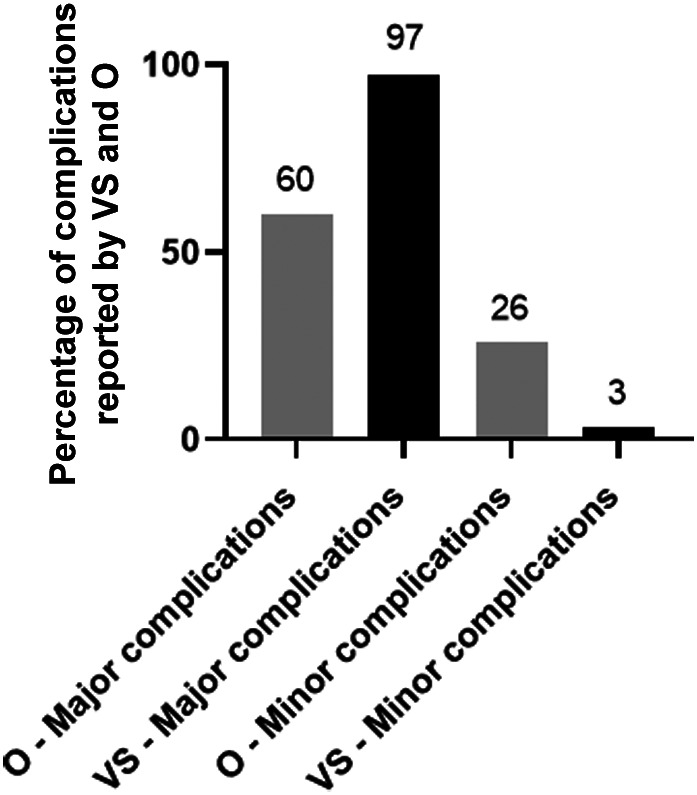
Percentage of complications reported by both veterinary surgeon and owners when the same number of dogs were compared. Complications were categorized according to severity (major and minor). O, owner. VS, veterinary surgeon.

**FIGURE 12:**
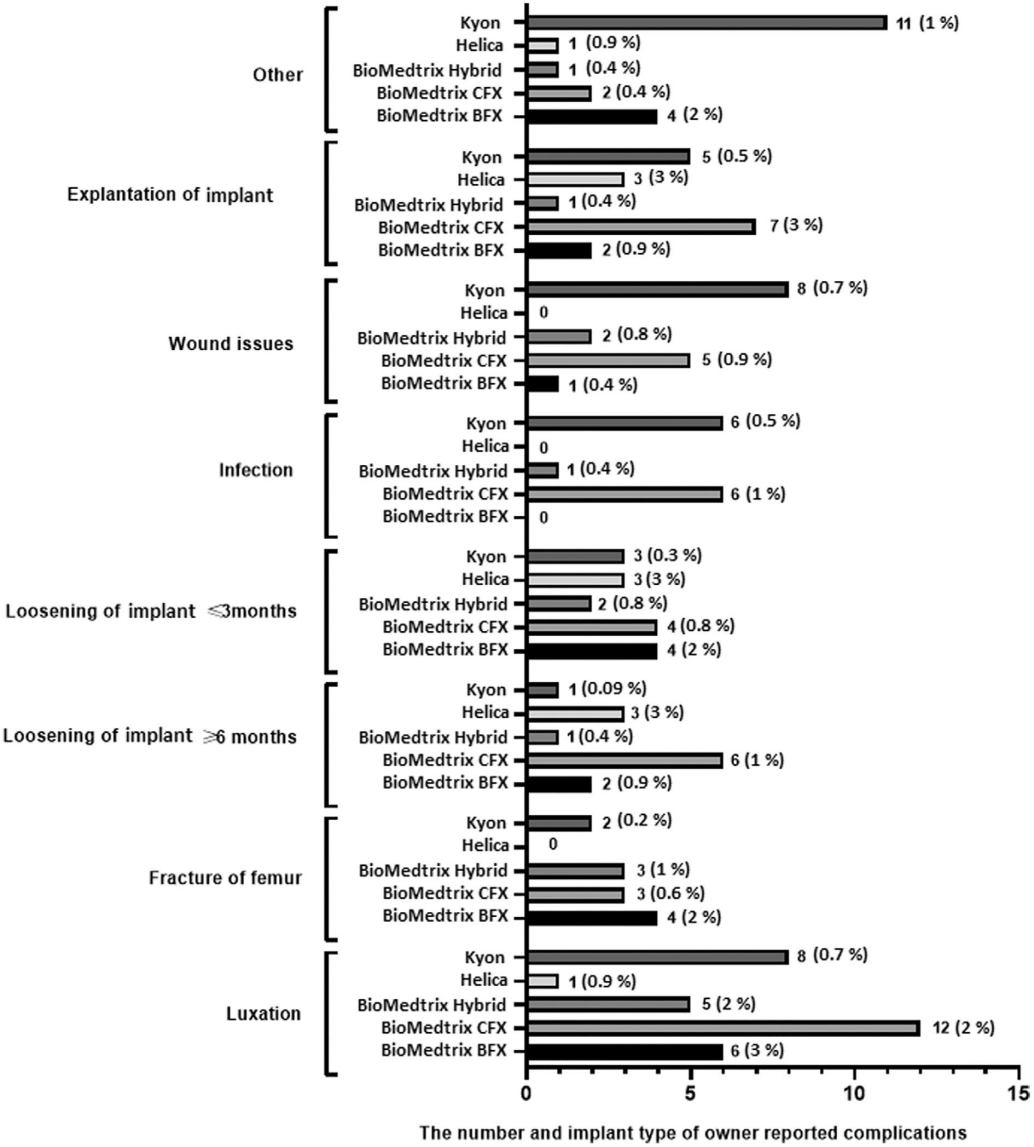
Owner reported complications related to different THR implant systems. THR, total hip replacement, CFX and BFX

The incidence of owner reported complications after THR was 23% (*n =* 107/461); however only 20% (*n =* 92/461) of the owners' complications contained sufficient data to analyze. The incidence of VS reported surgical complications was 7% (*n =* 31/461) for the cases for which the owners completed the online questionnaire. Cohen's kappa coefficient showed only moderate agreement of (0.44), (*P* < .001) between the incidence of complications reported by the owner and the VS. Weighted kappa indicated a moderate (0.5), (*P* < .001) agreement between the severity of complications reported by the owner and the VS. Owners reported minor complications (*n =* 28/107, 26%) and major complications (*n =* 64/107, 60%) compared to VS who reported 1/31 (3%) minor complication and 30/31 (97%) major complications (Figure 11). Forty‐three out of 461 owners (9%) showed that cases required 1 further operation, 17/461 (4%) cases required 2 further surgeries and 4/461 (1%) cases required 3 further surgeries subsequent to major complications. Overall, both VS and owner‐reported minor complications included issues with skin wound (n = 16/29), sciatic neuropraxia (*n =* 1/29), whilst major complications consisted of luxation (*n =* 36/94), infection of implant (*n =* 15/94), loosening of THR up to 3 months after surgery (*n =* 15/94), loosening of THR up to 6 months after surgery (*n =* 13/94), fracture of femur (*n =* 13/94), cup displacement (*n =* 8/94), sepsis (*n =* 5/94), aseptic loosening (*n =* 3/94) and other complications (*n =* 17/94). Forty‐three out of 107 (40%) owner‐reported complications were identified with Kyon implants, 30/107 (28%) with BioMedtrix CFX, 18/107 (17%) with BioMedtrix BFX, 9/107 (8%) with BioMedtrix Hybrid and 7/107 (6.5%) with Helica (Figure 12) (supplementary material 8). No associations were identified by univariable logistic regression analysis between bodyweight, age, sex, breed, indication for THR and the incidence of complication. However, on multivariable logistic regression, data showed that when femoral head and neck excision was the sole surgical indication, the type of implant used was associated with the complications (p = 0.031), with BioMedtrix BFX (OR 2.1, 95% CI 1.1‐4.4, p = 0.03) and Helica (OR 4.0, 95%CI 1.4‐11.3, p = 0.01) implants showing increased risk of complications.

## 4 DISCUSSION

This study reports clinical variables and complications following canine THRs registered on a multiuser CHR over a 10‐year period.

4.1

#### 4.1.1 Mortality over the 10‐year period

In our study, the total number of dogs reported to have died was 185/1852 and 94% of dogs that died or were euthanatized did so from causes or reasons unrelated to their THR. Four out of 11 (6%) dogs were euthanatized due to complications of the hip replacement or died suddenly due to a catastrophic complication. A 6% mortality rate associated with THR is substantially higher than that reported in human THR.^30^, ^31^ However, the 2 sectors cannot be directly compared since economic and welfare factors may play a role in decision‐making in veterinary clinical practice.^32^ Owners' ability to finance complications varies, and some dogs may have been euthanatized following a complication which may have been treatable but was not treated because of financial constraints or on grounds of animal welfare. Four out of 11 dogs in our study were euthanatized due to infection of the THR which may be due to a transient bacteremia upon implant insertion or biofilm development on the implant. Use of bone cement containing gentamycin has been shown to cause a statistically reduction in the rate of infection after total hip replacement in an experimental study.^33^, ^34^

#### 4.1.2 Implant systems

The CHR database consisted of 1852 dogs by June 2020, which is the highest number of dogs surveyed following THR to date.^15^, ^20^ The most frequent implant used in our study was Kyon (*n =* 1087/2375, 46%), which is different to previous CHR registry studies.^15^, ^20^ This appears to be due to the to the participation of recently enrolled veterinary practices in the BVOA‐UoL CHR, which use Kyon as their predominant implant system.

#### 4.1.3 Complications

The most frequent complications reported by VS and owners were luxation, femoral fracture and aseptic loosening (Figures 7, 12), as has been previously reported.^15^, ^20^ Femoral fractures were mostly associated with BioMedrix BFX implants in both VS and owner reported complications (Figures 7, 12), which agrees with previous literature, although this finding did not reach *P* = .05 in our study. When the sole surgical indication for canine THR was previous femoral head and neck excision, the risk of complications was significantly increased using BioMedtrix BFX and Helica implants (p = 0.031). This has not been previously reported with these implant systems but post THR complications such as aseptic loosening have been shown to occur with BioMedtrix CFX after previous femoral head and neck excision.^35^ These authors speculated that aseptic loosening of the acetabular cup might have been the result of inadequate cement fill of the acetabular socket, and poor bone‐cement interlock.^35^

#### 4.1.4 Veterinary surgeon and owner reporting complications

Owners reported a higher complication rate (23%) in comparison with VS (9%), with moderate agreement using a weighted kappa (k = 0.44). Complication rates reported by owners (23%) are similar to previously published reports from CHR.^15^ However, other studies reported that the owner‐reported complication rates were as low as 4.3% which may suggest a certain degree of unreliability in data reported by owners.^20^ Veterinary surgeon engagement with reporting complications can be difficult due to workload‐induced time constraints and perhaps a reluctance to report complications. Furthermore, the VS might have also been unaware of the complication that may not warrant referral back to a referral clinic, or if the surgeon who registered the case moved to a different clinic. The BVOA‐UoL CHR is a multi‐user database depending on individuals at participating veterinary clinics to input data on THR cases. Although we promote engagement with CHR through professional societies, the VS can be reluctant to enroll their cases and consistently report complications. We have recently employed a dedicated administrator to help improve future engagement and compliance. Another strategy to improve compliance in the veterinary sector would be to have an automated export of data from practice management systems.^36^

#### 4.1.5 Online owner‐administered questionnaire

Both an English and a nonvalidated translated version (Italian) of the owner‐administered questionnaire were sent to all registered owners. Our questionnaire response rate was 29%, which is less than previous CHR studies.^15^, ^20^ Two reminder e‐mails were sent to those owners who did not respond within 60 days of the first e‐mail. Previous studies have surveyed a smaller population (*n =* 170, and *n =* 136) within a shorter maximum follow‐up timeframe than this study with possibly better owner engagement. However, a 31% response rate has been reported as being acceptable in previously published large veterinary epidemiological studies.^37^ Most owners completed this survey within 12‐24 months (*n =* 103/461, 22%), and 24‐36 months (*n =* 105/461, 23%) after the initial THR, which agrees with a previous registry study showing that 85% of owners have replied to the owner‐based assessment questionnaire within 24 months of surgery.^20^ Owners may therefore remain engaged for a much shorter time following their dogs’ initial THR. It has been previously shown that response rates from patients can be improved by being younger in age, being able to speak the native language, being a new patient, having a longer wait time, and by being an immediate preoperative or postoperative patient.^37^

#### 4.1.6 Mobility

Our results showed an improvement between the overall mean of the preoperative and postoperative LOAD score (*P* < .0001), which has also been shown previously.^15^ The postoperative LOAD scores were better (*P* < .05) than preoperative scores up until the 82‐96 month follow‐up group although mean LOAD scores showed a tendency to be higher in the groups with longer follow up (Figure 9). This increase could be caused by a deterioration in the THR function but could also be caused by the presence of other musculoskeletal diseases. LOAD is a 3‐factor instrument and asks the owner to assess their dog's function at a “whole dog” level. Given that hip dysplasia and hip osteoarthritis are often bilateral but that the majority of dogs in this study had a unilateral THR, it is possible that OA in the contralateral hip joints may have contributed to this rise in LOAD scores postoperatively. Given that dogs with hip OA have increased risk of OA in other joints,^38^ this could also explain this finding.

#### 4.1.7 Limitations

Although this study includes the largest multiuser dataset reporting on the long‐term outcomes following canine THR, given the complication rates, the variance in type of complications and the number of different implant systems, there is still a likelihood of limited statistical power so the occurrence of type II errors cannot be ruled out. We found that VS were underreporting complications compared to owners, which may be due to the VS underreporting or owners overreporting complications. However, it was beyond the scope of this study to investigate possible causes of this discrepancy in reporting complications further. It might be due to referral VS predominantly treating major complications and overlooking minor complications; owners may seek treatment from first opinion practices for minor complications such as wound dehiscence. Furthermore, we were unable to establish whether increased complications were related to VS caseload given our confidentially agreement with participating VS. It has been well recognized that there is a learning curve associated with THR surgeries and a regular operative caseload is also advisable with this technique.

Due to large number of implant systems, we will endeavor to engage participating VS and owners in future with our dedicated administrative support enabling a larger sample size, which may be able to highlight any implant system issues. The response rate may have been influenced by the owner's perception of the success of the THR. The response rate also diminished with increased time since THR. This may suggest that not all owners have participated in the owner‐based assessment questionnaire.

## 5 CONCLUSION

Owner reported outcomes suggested that canine mobility improved after THR and that it was a safe and effective procedure. Previous femoral head and neck excision was associated with increased complications when a THR was performed using BioMedtrix BFX and Helica THR implant systems. Engagement and reporting of complications differed (*P* < .05) between VS and owners and in future should continue in a more reliable and consistent fashion.

## CONFLICT OF INTEREST

The authors declare no conflict of interest related to this report.

## Supporting information


Table S1

Table S2

Table S3

Table S4

Table S5

Table S6

Table S7

Table S8
