# Supplementary material for: Outcomes and complications reported from a multiuser canine hip replacement registry over a 10‐year period
Source: Vet Surg. 2022 Sep 5;52(2):196–208. doi: 10.1111/vsu.13885 (PMC10087566; doi:10.1111/vsu.13885)
Supplement: Supplementary file 1 — Table S1 [file VSU-52-196-s002.docx]

| Number of practices | Number of cases submitted |
| --- | --- |
| Practice No. 1 | 132 |
| Practice No. 2 | 129 |
| Practice No. 3 | 24 |
| Practice No. 4 | 5 |
| Practice No. 5 | 45 |
| Practice No. 6 | 132 |
| Practice No. 7 | 763 |
| Practice No. 8 | 4 |
| Practice No. 9 | 76 |
| Practice No. 10 | 49 |
| Practice No. 11 | 14 |
| Practice No. 12 | 32 |
| Practice No. 13 | 3 |
| Practice No. 14 | 1 |
| Practice No. 15 | 1 |
| Practice No. 16 | 38 |
| Practice No. 17 | 8 |
| Practice No. 18 | 32 |
| Practice No. 19 | 117 |
| Practice No. 20 | 4 |
| Practice No. 21 | 1 |
| Practice No. 22 | 1 |
| Practice No. 23 | 1 |
| Practice No. 24 | 2 |
| Practice No. 25 | 2 |
| Practice No. 26 | 26 |
| Practice No. 28 | 4 |
| Practice No. 29 | 157 |
| Practice No. 30 | 85 |
