# Supplementary material for: Outcomes and complications reported from a multiuser canine hip replacement registry over a 10‐year period
Source: Vet Surg. 2022 Sep 5;52(2):196–208. doi: 10.1111/vsu.13885 (PMC10087566; doi:10.1111/vsu.13885)
Supplement: Supplementary file 2 — Table S2 [file VSU-52-196-s005.docx]

| Cause of death | Number of dead dogs |
| --- | --- |
| Complications associated with total hip replacement | 7 |
| Cardiac disease | 9 |
| Old age | 9 |
| Neoplasia | 31 |
| Musculoskeletal disease (other than hip-related) | 13 |
| Not specified | 29 |
| Owner did not report cause of death | 58 |
| Dog had more than one cause of death | 5 |
| Euthanized due to complications of the hip | 4 |
| Euthanized due to cardiac disease | 2 |
| Euthanized due to neoplasia | 3 |
| Euthanized due to musculoskeletal disease | 1 |
| Euthanized due to other causes | 8 |
| Unknown reason for euthanasia | 6 |
| Subtotal | 185 |
