# Supplementary material for: Outcomes and complications reported from a multiuser canine hip replacement registry over a 10‐year period
Source: Vet Surg. 2022 Sep 5;52(2):196–208. doi: 10.1111/vsu.13885 (PMC10087566; doi:10.1111/vsu.13885)
Supplement: Supplementary file 3 — Table S3 [file VSU-52-196-s007.docx]

| Breed | Number | Percentage |
| --- | --- | --- |
| Labrador Retriever | 376 | 20.3 |
| Crossbreed | 280 | 15.1 |
| German Shepherd | 238 | 12.9 |
| Border Collie | 136 | 7.3 |
| Golden Retriever | 106 | 5.7 |
| Rottweiler | 66 | 3.6 |
| Other | 48 | 2.6 |
| Bernes Mountain dog | 41 | 2.2 |
| English Springer Spaniel | 36 | 1.9 |
| Newfoundland | 28 | 1.5 |
| West Highland White Terrier | 26 | 1.4 |
| Cocker Spaniel | 26 | 1.4 |
| Cane Corso | 26 | 1.4 |
| English Setter | 25 | 1.3 |
| Labradoodle | 22 | 1.2 |
| English Cocker Spaniel | 21 | 1.1 |
| Otterhound | 17 | 0.9 |
| Akita | 17 | 0.9 |
| Mastiff | 14 | 0.8 |
| Airedale Terrier | 14 | 0.8 |
| Pasrot Maremmano Abruzese | 14 | 0.8 |
| Staffordshire Bull Terrier | 13 | 0.7 |
| Cavalier King Charles Spaniel | 13 | 0.7 |
| Boxer | 13 | 0.7 |
| Jack Russell Terrier | 13 | 0.7 |
| Chow Chow | 10 | 0.5 |
| Not specified | 10 | 0.5 |
| Pug | 9 | 0.5 |
| Samoyed | 8 | 0.4 |
| American Bulldog | 8 | 0.4 |
| Border Terrier | 7 | 0.4 |
| Cekcoslohakian Wolfdog | 7 | 0.4 |
| Visla | 7 | 0.4 |
| Yorkshire Terrier | 7 | 0.4 |
| Weimaraner | 6 | 0.3 |
| Siberian Husky | 6 | 0.3 |
| Old English sheepdog | 6 | 0.3 |
| Alaskan Malamute | 6 | 0.3 |
| Lagotto Romagnolo | 6 | 0.3 |
| Lurcher | 6 | 0.3 |
| Dogue De Bordeaux | 6 | 0.3 |
| Beagle | 5 | 0.3 |
| Belgian Malinois | 5 | 0.3 |
| Briard | 5 | 0.3 |
| Pointer | 5 | 0.3 |
| Shih Tzu | 5 | 0.3 |
| Tibetan Terrier | 5 | 0.3 |
| Swiss Shepherd | 4 | 0.2 |
| Saint Bernard | 4 | 0.2 |
| Pitbull | 4 | 0.2 |
| Flat Coated Retriever | 4 | 0.2 |
| Caucasian Shepherd | 4 | 0.2 |
| Bearded Collie | 4 | 0.2 |
| Welsh Springer Spaniel | 3 | 0.2 |
| Shetland Sheepdog | 3 | 0.2 |
| Rhodesian Ridgeback | 3 | 0.2 |
| Poodle | 3 | 0.2 |
| Pomeranian | 3 | 0.2 |
| Leonberger | 3 | 0.2 |
| Gordon Setter | 3 | 0.2 |
| Doberman Pinscher | 3 | 0.2 |
| Northern Inuit Dog | 3 | 0.2 |
| Chihuahua | 3 | 0.2 |
| Bichon Frise | 2 | 0.1 |
| Central Asian Shepherd | 2 | 0.1 |
| French Bulldog | 2 | 0.1 |
| Griffon Brussels | 2 | 0.1 |
| Affenpinscher | 2 | 0.1 |
| Afghan Hound | 2 | 0.1 |
| Clumber Spaniel | 3 | 0.2 |
| Bull Terrier | 2 | 0.1 |
| Rough Collie | 3 | 0.2 |
| Anatolian Shepherd | 2 | 0.1 |
| Bergamasco Shepherd | 2 | 0.1 |
| Subtotal | 1852 | 100 |
