# Supplementary material for: Outcomes and complications reported from a multiuser canine hip replacement registry over a 10‐year period
Source: Vet Surg. 2022 Sep 5;52(2):196–208. doi: 10.1111/vsu.13885 (PMC10087566; doi:10.1111/vsu.13885)
Supplement: Supplementary file 4 — Table S4 [file VSU-52-196-s006.docx]

1. CFX implant size for first and second hip operations

| CFX head size at first hip operation | CFX femoral stem size at first hip operation | CFX acetabular cup size at first hip operation | CFX head size at second hip operation | CFX femoral stem size at second hip operation | CFX acetabular cup size at second hip operation |
| --- | --- | --- | --- | --- | --- |
| 12mm + 0 Femoral Head (n= 11) | Size 1 +0 (n= 5) | Size 4 (n= 1) | 12mm + 0 Femoral Head (n= 1) | Size 2 (n= 2) | Size 12 (n= 1) |
| 12mm + 3 Femoral Head (n= 36) | Size 1 +2 (n= 1) | Size 10 (n= 10) | 12mm + 3 Femoral Head (n= 6) | Size 3 (n= 9) | Size 14 (n= 4) |
| 12mm + 6 Femoral Head (n= 2) | Size 1 +4 (n= 1) | Size 12 (n= 35) | 14mm + 1 Femoral Head (n= 2) | Size 4 (n= 7) | Size 16 (n= 6) |
| 13mm + 1 Femoral Head (n= 1) | Nano (n= 3) | Size 14 (n= 59) | 14mm + 3 Femoral Head (n= 3) | Size 4/5 (n= 1) | Size 18 (n= 7) |
| 14mm + 1 Femoral Head (n= 13) | Size 2 (n= 63) | Size 16 (n= 31) | 14mm + 5 Femoral Head (n= 3) | Size 5 (n= 19) | Size 20 (n= 9) |
| 14mm + 1 Femoral Head (n= 2) | Size 3 (n= 62) | Size 18 (n= 48) | 17mm + 0 Femoral Head (n= 19) | Size 6 (n= 12) | Size 23 (n= 13) |
| 14mm + 3 Femoral Head (n= 3) | Size 4 (n= 50) | Size 20 (n= 40) | 17mm + 3 Femoral Head (n= 15) | Size 7 (n= 10) | Size 25 (n= 16) |
| 14mm + 5 Femoral Head (n= 3) | Size 4/5 (n= 7) | Size 23 (n= 104) | 17mm + 6 Femoral Head (n= 7) | Size 8 (n= 6) | Size 27 (n= 10) |
| 17mm + 0 Femoral Head (n= 19) | Size 5 (n= 113) | Size 25 (n= 69) | 8mm + 0 Femoral Head (n= 6) | Size 9 (n= 2) | Size 29 (n= 2) |
| 17mm + 3 Femoral Head (n= 15) | Size 6 (n= 70) | Size 27 (n= 28) | 8mm + 2 Femoral Head (n= 4) |  |  |
| 17mm + 6 Femoral Head (n= 7) | Size 7 (n= 33) | Size 29 (n= 6) |  |  |  |
| 8mm + 0 Femoral Head (n= 6) | Size 8 (n= 20) |  |  |  |  |
| 8mm + 2 Femoral Head (n= 4) | Size 9 (n= 7) |  |  |  |  |
| 8mm + 5 Femoral Head (n= 4) |  |  |  |  |  |

1. BFX implant size for first and second hip operations

| BFX head size at first hip operation | BFX femoral stem size at first hip operation | BFX acetabular cup size at first hip operation | BFX head size at second hip operation | BFX femoral stem size at second hip operation | BFX acetabular cup size at second hip operation |
| --- | --- | --- | --- | --- | --- |
| 12mm + 0 Femoral Head (n= 2) | Size 2 (n= 1) | Size 12 (n= 1) | 12mm + 3 Femoral Head (n= 1) | Size 4 (n= 1) | Size 20 (ID 13mm) (n= 2) |
| 12mm + 3 Femoral Head (n= 1) | Size 4 (n= 6) | Size 20 (n= 1) | 13mm + 5 Femoral Head (n= 2) | Size 5 (n= 1) | Size 22 (ID 14mm) (n= 6) |
| 13mm + 1 Femoral Head (n= 6) | Size 5 (n= 9) | Size 20 (ID 12mm) (n= 6) | 14mm + 1 Femoral Head (n= 3) | Size 6 (n= 4) | Size 24 (n= 3) |
| 13mm + 3 Femoral Head (n= 4) | Size 6 (n= 26) | Size 20 (ID 12mm) (n= 6) | 14mm + 3 Femoral Head (n= 1) | Size 7 (n= 6) | Size 24 (ID 17mm) (n= 11) |
| 13mm + 5 Femoral Head (n= 5) | Size 7 (n= 55) | 22 (ID 12mm) (n= 3) | 14mm + 5 Femoral Head (n= 2) | Size 8 (n= 16) | Size 28 (ID 17mm) (n= 2) |
| 14mm + 1 Femoral Head (n= 13) | Size 8 (n= 35) | Size 22 (ID 14mm) (n= 27) | 17mm + 0 Femoral Head (n= 8) | Size 9 (n= 4) | Size 30 (ID 22mm) (n= 1) |
| 14mm + 3 Femoral Head (n= 3) | Size 9 (n= 28) | Size 24 (n= 5) | 17mm + 3 Femoral Head (n= 11) | Size 10 (n= 2) |  |
| 14mm + 5 Femoral Head (n= 9) | Size 10 (n= 18) | Size 24 (ID 17mm) (n= 42) | 17mm +6 Femoral Head (n= 4) | Size 11 (n= 1) |  |
| 17mm + 0 Femoral Head (n= 55) | Size 11 (n= 6) | Size 26 (n= 2) | 17mm + 9 Femoral Head (n= 1) |  |  |
| 17mm + 3 Femoral Head (n= 44) | Size 12 (n= 2) | Size 26 (ID 17mm) (n= 37) | 22mm + 0 Femoral Head (n= 1) |  |  |
| 17mm + 6 Femoral Head (n= 18) |  | Size 28 (n= 4) |  |  |  |
| 17mm + 9 Femoral Head (n= 5) |  | Size 28 (ID 17mm) (n= 13) |  |  |  |
| 22mm + 0 Femoral Head (n= 3) |  | Size 30 (n= 2) |  |  |  |
| 22mm + 3 Femoral Head (n= 2) |  | 30 (ID 17mm) (n= 3) |  |  |  |
| 22mm + 6 Femoral Head (n= 1) |  | 30 (ID 22mm) (n= 5) |  |  |  |
| 8mm + 0 Femoral Head (n= 2) |  |  |  |  |  |

1. Hybrid CFX femoral implant and BFX acetabular cup size for both hip operations

| Hybrid CFX/ BFX head size at first hip operation | CFX femoral implant size at first hip operation | BFX acetabular cup size at first hip operation | Hybrid CFX/ BFX head size at second hip operation | CFX femoral implant size at second hip operation | BFX acetabular cup size at second hip operation |
| --- | --- | --- | --- | --- | --- |
| 14mm + 3 Femoral Head (n= 10) | Size 2 (n= 1) | Size 20mm (n= 4) | 12mm + 0 Femoral Head (n= 1) | Size 4 (n= 2) | Size 20 (ID 12mm) (n= 1) |
| 12mm + 0 Femoral Head (n= 8) | Size 4 (n= 12) | Size 20 (ID 12mm) (n= 5) | 12mm + 3 Femoral Head (n= 1) | Size 5 (n= 8) | Size 20 (ID 13mm) (n= 1) |
| 12mm + 3 Femoral Head (n= 6) | Size 4/5 (n= 4) | Size 20 (ID 13mm) (n= 4) | 13mm + 1 Femoral Head (n= 1) | Size 6 (n= 16) | Size 22mm (ID 14mm) (n= 1) |
| 14mm + 1 Femoral Head (n= 8) | Size 5 (n= 33) | Size 22 (ID 12mm) (n= 2) | 14mm + 3 Femoral Head (n= 1) | Size 7 (n= 10) | Size 24mm (n= 8) |
| 22mm + 3 Femoral Head (n= 3) | Size 6 (n= 66) | Size 22 (ID 14mm) (n= 24) | 14mm + 5 Femoral Head (n= 1) | Size 8 (n= 5) | Size 24 (ID 17mm) (n= 7) |
| 14mm + 5 Femoral Head (n= 9) | Size 7 (n= 42) | Size 24mm (n= 13) | 17mm + 0 Femoral Head (n= 18) | Size 9 (n= 3) | Size 26 (ID17mm) (n= 12) |
| 17mm + 13 Femoral Head (n= 1) | Size 8 (n= 23) | Size 24 (ID 17mm) (n= 30) | 17mm + 3 Femoral Head (n= 10) |  | Size 28 (n= 1) |
| 17mm + 0 Femoral Head (n= 68) | Size 9 (n= 8) | Size 26mm (n= 8) | 17mm +6 Femoral Head (n= 8) |  | Size 28 (ID 17mm) (n= 4) |
| 17mm + 3 Femoral Head (n= 52) | Size 10 (n= 5) | Size 26 (ID 17mm) (n= 19) | 22mm + 0 Femoral Head (n= 1) |  | Size 30 (ID 17mm) (n= 1) |
| 17mm + 6 Femoral Head (n= 16) |  | Size 28 (n= 6) |  |  | Size 30 (ID 22mm) (n= 2) |
| 17mm + 9 Femoral Head (n= 5) |  | Size 28 (ID 17mm) (n= 16) |  |  |  |
| 22mm + 0 Femoral Head (n= 3) |  | Size 30mm (n= 3) |  |  |  |
| 8mm + 2 Femoral Head (n= 1) |  | Size 30 (ID 22mm) (n= 1) |  |  |  |
| 13mm + 1 Femoral Head (n= 1) |  | Size 30 (ID 17mm) (n= 3) |  |  |  |
| 8mm + 0 Femoral Head (n= 1) |  | Size 32 (n= 1) |  |  |  |
|  |  | Size 32 (ID 17mm) (n= 1) |  |  |  |
|  |  | Size 32 (ID 22mm) (n= 3) |  |  |  |
|  |  | Size 34 (ID 17mm) (n= 1) |  |  |  |

1. Hybrid BFX femoral implant and CFX acetabular cup implant size for first and second hip operations

| Hybrid CFX/ BFX head size at first hip operation | BFX femoral implant size at first hip operation | CFX acetabular cup size at first hip operation | Hybrid CFX/ BFX head size at second hip operation | BFX femoral implant size at second hip operation | CFX acetabular cup size at second hip operation |
| --- | --- | --- | --- | --- | --- |
| 12mm + 0 Femoral Head (n= 3) | Size 18 (n= 4) | Size 4 (n= 4) | 14mm + 3 Femoral Head (n= 1) | Size 5 (n= 1) | Size 20 (n= 1) |
| 12mm + 3 Femoral Head (n= 1) | Size 19 (n=4) | Size 5 (n=4) |  |  |  |
| 13mm + 1 Femoral Head (n= 1) | Size 23 (n=3) | Size 6 (n= 2) |  |  |  |
| 13mm + 5 Femoral Head (n= 3) |  | Size 8 (n= 1) |  |  |  |
| 17mm + 3 Femoral Head (n= 3) |  | Size 9 (n= 1) |  |  |  |
| 17mm + 0 Femoral Head (n= 1) |  |  |  |  |  |

1. Kyon implant sizes for first and second hip operations

| Kyon femoral stem size at first hip operation | Kyon acetabular cup size at first hip operation | Kyon femoral head and neck size at first hip operation | Kyon femoral stem size at second hip operation | Kyon acetabular cup size at second hip operation | Kyon femoral head and neck size at second hip operation |
| --- | --- | --- | --- | --- | --- |
| X Small plus 4 screws (n= 76) | XX Small (n= 61) | X Short (n= 44) | X Small plus 4 screws (n= 27) | XX Small (n= 22) | X Short (n= 10) |
| Small plus 4 screws (n= 188) | X Small (n= 193) | Short (n= 387) | Small plus 4 screws (n= 85) | X Small (n= 89) | Short (n= 184) |
| Medium plus 5 screws (n= 280) | Small (n= 289) | Short and big head (n= 59) | Medium plus 5 screws (n= 128) | Small (n= 136) | Short and big head (n= 32) |
| Large plus 5 screws (n= 188) | Medium (n= 184) | Medium (n= 69) | Large plus 5 screws (n= 84) | Medium (n= 77) | Medium (n=174) |
| X Large plus 6 screws (n= 19) | Large (n= 13) | Long and big head (n= 5) | X Large plus 6 screws (n= 11) | Large (n= 10) | Long (n= 2) |
|  |  | X Long and big head (n= 2) |  |  | Long and big head (n= 2) |

1. Helica implant sizes for first hip operation

| Helica femoral stem length at first hip operation | Helica femoral stem diameter at first hip operation | Helica acetabular cup diameter at first hip operation | Helica head and neck size at first hip operation | Helica head size only at first hip operation |
| --- | --- | --- | --- | --- |
| Size 41 mm (n= 3) | Size 8 mm (n= 20) | Size 32 mm (n= 3) | X Large (n= 4) | Size 15 mm (n= 11) |
| Size 39 mm (n= 2) | Size 9 mm (n= 6) | Size 30 mm (n= 3) | Large (n= 14) | Size 18 mm (n= 37) |
| Size 37 mm (n= 4) | Size 10 mm (n= 26) | Size 28 mm (n= 44) | Medium (n= 23) |  |
| Size 35 mm (n= 29) | Size 11 mm (n= 7) | Size 24 mm (n= 32) | Small (n= 33) |  |
| Size 32 mm (n= 10) | Size 12 mm (n= 5) | Size 22 mm (n= 1) |  |  |
| Size 31 mm (n= 28) |  | Size 20 mm (n= 8) |  |  |
| Size 28 mm (n= 5) |  |  |  |  |
| Size 26 mm (n= 24) |  |  |  |  |

1. Helica implant sizes for Second hip operation

| Helica femoral stem length at second hip operation | Helica femoral stem diameter at second hip operation | Helica acetabular cup diameter at second hip operation | Helica head and neck size at second hip operation | Helica head size only at second hip operation |
| --- | --- | --- | --- | --- |
| Size 41 mm (n= 1) | Size 11 mm (n= 3) | Size 30 mm (n= 1) | Medium (n= 2) | Size 15 mm (n= 2) |
| Size 37 mm (n= 2) | Size 10 mm (n= 2) | Size 28 mm (n= 2) | Small (n= 11) | Size 18 mm (n= 8) |
| Size 35 mm (n= 2) | Size 8 mm (n= 9) | Size 26 mm (n= 4) |  |  |
| Size 32 mm (n= 1) |  | Size 24 mm (n= 5) |  |  |
| Size 31 mm (n= 6) |  | Size 22 mm (n= 2) |  |  |
| Size 26 mm (n= 2) |  |  |  |  |
