# Supplementary material for: Outcomes and complications reported from a multiuser canine hip replacement registry over a 10‐year period
Source: Vet Surg. 2022 Sep 5;52(2):196–208. doi: 10.1111/vsu.13885 (PMC10087566; doi:10.1111/vsu.13885)
Supplement: Supplementary file 5 — Table S5 [file VSU-52-196-s004.docx]

| Complication | Complication classification | Implant system | Action required |
| --- | --- | --- | --- |
| Acetabular cup displacement | Major | Biomedtrix BFX | Revision with new implant. Replaced with new BFX acetabular cup size and new BFX/CFX head size. |
| Acetabular cup displacement | Major |  | Conservative treatment |
| Acetabular cup displacement | Major | Biomedtrix CFX | Explantation and femoral head and neck excision. |
| acetabular cup displacement | Major |  | Explantation |
| Acetabular cup displacement | Major |  | Revision with new implant (also had prosthesis breakage) |
| Acetabular cup displacement | Major | Helica | Revision with new implant. Replaced with new acetabular cup size |
| Acetabular cup displacement | Major |  | Revision with new implant. Replaced with new acetabular cup size |
| Acetabular cup displacement | Major | Biomedtrix Hybrid | Revision with new implant. Replaced with new acetabular cup size |
| Acetabular cup displacement | Major | Kyon | Revision with new implant |
| Acetabular cup displacement | Major | Kyon | Revision with new implant |
| Acetabular cup displacement | Major | Kyon | Revision with new implant |
| Acetabular cup displacement | Major |  | Revision with new implant and had a new ceramic heard |
| Acetabular cup displacement | Major | Kyon | Revision with new implant. Revised with new Kyon acetabular cup size and new long Kyon head-neck size |
| Acetabular cup displacement | Major | Biomedtrix Hybrid | Revision with new implant |
| Acetabular cup displacement | Major | Biomedtrix CFX | Revision with same implant |
| Acetabular cup displacement | Major | Biomedtrix CFX | Revision with same implant |
| Acetabular cup displacement | Minor | Biomedtrix BFX | Revision with new Helica implant. |
| Acetabular cup displacement | Major |  | Amputation (also had aseptic loosening) |
| Acetabular cup displacement | Major |  | Revision with new implant |
| Acetabular dorsal rim fracture | Major | Biomedtrix CFX | Explantation |
| Acetabular fracture | Major | Biomedtrix Hybrid | Fracture repair (Cerclage) and buttress locking compression plate |
| Acetabular fracture | Major | Biomedtrix BFX | Repair fracture |
| Acetabular fracture | Major | Biomedtrix BFX | Repair fracture with locking plate and screws |
| Acetabular fracture | Major | Biomedtrix CFX | No action reported |
| Acetabular cup subsidence | Major | Biomedtrix CFX | No action reported |
| Aseptic loosening | Major |  | Amputation (also had acetabular cup displacement) |
| Aseptic loosening | Major | Kyon | Revised with new implant. Replaced with new Kyon acetabular cup |
| Aseptic loosening | Catastrophic |  | Euthanasia |
| Aseptic loosening | Major | Helica | Explantation of femoral stem |
| Aseptic loosening | Major |  | Explantation of femoral stem |
| Aseptic loosening | Major | Helica | Explantation of femoral stem but not acetabular cup |
| Aseptic loosening | Major | Helica | Explantation of femoral stem but not acetabular cup |
| Aseptic loosening | Major | Biomedtrix CFX | Explantation |
| Aseptic loosening | Major | Biomedtrix CFX | Explantation |
| Aseptic loosening | Major | Helica | Explantation |
| Aseptic loosening | Major | Kyon | Explantation |
| Aseptic loosening | Major |  | Explantation |
| Aseptic loosening | Major | Helica | Explantation. |
| Aseptic loosening | Major | Helica | Revision with new implant |
| Aseptic loosening | Major | Helica | Revision with new implant |
| Aseptic loosening | Major | Helica | Revision with new implant |
| Aseptic loosening | Major |  | Revision with new implant |
| Aseptic loosening | Major | Kyon | Revision with new implant. Replaced With a new acetabular cup with separate shell and liner. Replaced also with a new head size (also had prosthesis related sepsis) |
| Aseptic loosening | Major | Kyon | Revision with new implant |
| Aseptic loosening | Major | Biomedtrix CFX | Revision with same implant |
| Aseptic loosening | Major | Helica | Revision with same implant |
| Aseptic loosening | Major | Helica | Revision with same implant |
| Aseptic loosening | Major |  | Revision with same implant |
| Aseptic loosening | Major | Helica | Revision with same implant |
| Aseptic loosening | Major |  | Closed reduction and explantation (also had aseptic loosening and luxation |
| Aseptic loosening | Major | Helica | Revision with same implant and explantation (also had prosthesis related sepsis) |
| Aseptic loosening of the femoral head and stem | Major | Biomedtrix CFX | revision attempted and failed. explantation (n=2) |
| Aseptic loosening of the femoral stem | Major | Biomedtrix CFX | Revision with new implant(n=2) and explantation (also had extraosseous cement granuloma) |
| Aseptic loosening of the femoral stem | Major |  | Explantation (also had luxation) |
| Aseptic loosening of the femoral stem | Major | Helica | Explantation and excision arthroplasty |
| Breakage of femoral stem prosthesis | Major |  | Explantation of femoral stem but not acetabular cup |
| Extraosseous cement granuloma | Major | Biomedtrix CFX | Revision with new implant(n=2) and explantation (also had aseptic loosening) |
| Femoral fissure | Major | Biomedtrix Hybrid | Fissure fracture repair (Cerclage) |
| femoral fissure fracture | Major | Biomedtrix CFX | Revisison with new implant. Replaced with new CFX femoral stem size. Fissure fracture repaired (Cerclage). |
| Femoral fissure fracture | Major | Biomedtrix BFX | Fissure fracture repair (Cerclage) |
| Femoral fissure fracture | Major | Biomedtrix CFX | Fissure fracture repair (Cerclage) |
| Femoral fissure fracture | Major | Biomedtrix BFX | Fissure fracture repair (Cerclage) |
| Femoral fissure fracture | Major | Biomedtrix BFX | Explantation and femoral fissure fracture repair (Cerclage) |
| Femoral fissure fracture | Major |  | Closed reduction and explantation |
| Femoral fissure fracture | Major |  | No action reported |
| Femoral fissure fracture | Major | Biomedtrix CFX | Fissure fracture repair (Cerclage) |
| Femoral fissure fracture | Major | Kyon | Repair fracture |
| Femoral fissure fracture | Major |  | Repair fracture |
| Femoral fissure fracture | Major | Kyon | Repair fracture |
| Femoral fissure fracture | Major | Biomedtrix BFX | N/A |
| Femoral fissure fracture | Major | Biomedtrix Hybrid | Fissure fracture repair (Cerclage) |
| Femoral fissure fracture | Major | Biomedtrix BFX | Fissure fracture repair (Cerclage wires) |
| Femoral fracture | Catastrophic | Kyon | Euthanasia |
| Femoral fracture | Catastrophic | Helica | Euthanasia |
| Femoral fracture | Major | Biomedtrix CFX | femoral head and neck excision |
| Femoral fracture | Major | Biomedtrix CFX | Fracture repair with internal fixation |
| Femoral fracture | Major | Biomedtrix BFX | Explantation |
| Femoral fracture | Major | Biomedtrix BFX | Repair fracture |
| Femoral fracture | Major | Biomedtrix BFX | Repair fracture |
| Femoral fracture | Major | Biomedtrix CFX | Repair fracture |
| Femoral fracture | Major | Biomedtrix CFX | Repair fracture |
| Femoral fracture | Major | Biomedtrix Hybrid | Repair fracture |
| Femoral fracture | Major | Biomedtrix Hybrid | Repair fracture |
| Femoral fracture | Major | Biomedtrix Hybrid | Repair fracture |
| Femoral fracture | Major | Kyon | Repair fracture |
| Femoral fracture | Major | Kyon | Repair fracture |
| Femoral fracture | Major |  | Repair fracture |
| Femoral fracture | Major | Biomedtrix Hybrid | Repair fracture |
| Femoral fracture | Major | Biomedtrix BFX | Revision with new CFX acetabular cup and femoral stem size |
| Femoral fracture | Major | Biomedtrix CFX | Revision with same implant |
| Femoral fracture | Major | Kyon | N/A |
| Femoral fracture | Major |  | multiple revisions |
| Femoral fracture | Major |  | Explantation |
| Femoral fracture | Major | Biomedtrix BFX | Femoral fracture repair (Cerclage) |
| Femoral fracture | Major | Biomedtrix BFX | Fracture repair (Cerclage) |
| Femoral fracture | Major | Biomedtrix Hybrid | Repair fracture (Cerclage). |
| Femoral fracture | Major | Biomedtrix Hybrid | Repair fracture |
| Femoral pain | Minor | Biomedtrix CFX | No action reported |
| Femoral subsidence | Major | Biomedtrix BFX | Open reduction + Revision with new implant. Replaced with new BFX acetabular cup size. Cerclage wires placed around proximal femur (also had luxation) |
| Femoral subsidence | Major | Biomedtrix BFX | Conservative treatment |
| Femoral subsidence | Major |  | Explantation of femoral stem |
| Femoral subsidence | Major | Biomedtrix BFX | Revision with new implant. Replace with new CFX femoral stem size |
| Hip luxation | Major | Biomedtrix CFX | Closed reduction and acetabular cup repositioned |
| Intra-operative cardiac arrest | Catastrophic | Biomedtrix Hybrid | Died |
| Lateral patella luxation | Major |  | Block recession sulcoplasty |
| Luxation | Major | Biomedtrix BFX | Stabilisation with Ehmer sling technique |
| Luxation | Major | Kyon | Explantation |
| Luxation | Major |  | Closed reduction and explantation (also had luxation and femoral fissure fracture) |
| Luxation | Major | Biomedtrix BFX | Ilio-femoral suture was placed |
| Luxation | Major |  | Revision with new implant. Replaced with new CFX acetabular cup size |
| Luxation | Major | Biomedtrix Hybrid | Revision |
| Luxation | Major | Kyon | Revision with new implant. Acetabular cup repositioned into a more closed position and replaced with new long Kyon head-neck size |
| Luxation | Major | Biomedtrix BFX | Revision with new implant and open reduction for explantation (also had prosthesis related sepsis) |
| Luxation | Major | Biomedtrix Hybrid | Revision with new implant (n=2) and open reduction for explantation (also had prosthesis related sepsis) |
| Luxation | Major | Kyon | Revision with new implant and open reduction |
| Luxation | Major |  | Revision with same implant and open reduction |
| Luxation | Major | Biomedtrix BFX | N/A |
| Luxation | Major | Biomedtrix BFX | Open reduction + Revision with new implant. Replaced with new BFX acetabular cup size. Cerclage wires placed around proximal femur (also had femoral subsidence) |
| Luxation | Major | Biomedtrix BFX | Open reduction |
| Luxation | Major | Helica | closed reduction and stabilisation with Ehmer sling technique. |
| Luxation | Major | Biomedtrix CFX | Closed reduction and Stabilisation with dorsal sling technique |
| Luxation | Major |  | Closed reduction and explantation (also had aseptic loosening and femoral fissure fracture) |
| Luxation | Major | Biomedtrix CFX | Explantation for open reduction |
| Luxation | Major | Biomedtrix CFX | Explantation for open reduction |
| Luxation | Major | Biomedtrix CFX | Explantation for open reduction |
| Luxation | Major |  | Closed reduction and explantation |
| Luxation | Major | Biomedtrix BFX | Explantation |
| Luxation | Major |  | Explantation (also had aseptic loosening) |
| Luxation | Major | Biomedtrix Hybrid | Open reduction and new femoral head size replaced |
| Luxation | Major | Kyon | Open reduction and new long Kyon head-nice size replaced |
| Luxation | Major |  | Open reduction and capsullorraphy |
| Luxation | Major | Helica | Open reduction, Ilio/femoral suture and capsular imbrication |
| Luxation | Major | Helica | Open reduction, Ilio-femoral suture was placed and acetabular cup was repositioned |
| Luxation | Major | Helica | Open reduction and Ilio-femoral suture was placed |
| Luxation | Major | Biomedtrix BFX | Open reduction and Ilio-femoral suture was placed |
| Luxation | Major | Biomedtrix BFX | Excision of new bone formation and closed reduction |
| Luxation | Major | Kyon | Open reduction and new Kyon head-neck size replaced |
| Luxation | Major | Biomedtrix BFX | Open reduction |
| Luxation | Major | Helica | Open reduction and ileofemoral suture was placed |
| Luxation | Major |  | Open reduction, Stabilised with dorsal sling technique |
| Luxation | Major | Biomedtrix CFX | Physiotherapy |
| Luxation | Major | Biomedtrix CFX | Open reduction and revision with new acetabular cup size |
| Luxation | Major | Biomedtrix BFX | Open reduction, revision with new Helica implant and acetabular cup repositioned into a more closed position |
| Luxation | Major |  | Open reduction, revision with new acetabular cup and head size |
| Luxation | Major | Biomedtrix CFX | Open reduction and revision with new implant |
| Luxation | Major | Biomedtrix CFX | Open reduction and revision with new implant |
| Luxation | Major | Biomedtrix CFX | Open reduction and revision with new implant |
| Luxation | Major | Biomedtrix CFX | Open reduction and revision with new implant |
| Luxation | Major | Kyon | Open reduction and revision with new implant |
| Luxation | Major | Kyon | Open reduction and revision with new implant |
| Luxation | Major | Kyon | Open reduction and revision with new implant |
| Luxation | Major | Biomedtrix CFX | Open reduction and revision with new implant |
| Luxation | Major | Biomedtrix BFX | Open reduction, revision with new CFX acetabular cup. Acetabular cup repositioned into a more closed position |
| Luxation | Major |  | Open reduction, acetabular cup repositioned into a more closed position and revision with new longer BFX/CFX head-neck size |
| Luxation | Major | Biomedtrix Hybrid | Revision with new implant |
| Luxation | Major |  | Revised with new acetabular cup size and open reduction |
| Luxation | Major | Kyon | Revision with new implant and closed reduction |
| Luxation | Major | Biomedtrix BFX | Revision with same implant and open reduction |
| Luxation | Major | Biomedtrix BFX | Revision with same implant and open reduction |
| Luxation | Major | Biomedtrix BFX | Revision with same implant and open reduction |
| Luxation | Major | Biomedtrix CFX | Revision with same implant and open reduction |
| Luxation | Major | Biomedtrix CFX | Revision with same implant and open reduction |
| Luxation | Major | Biomedtrix CFX | Revision with same implant and open reduction |
| Luxation | Major | Biomedtrix CFX | Revision with same implant and open reduction |
| Luxation | Major | Biomedtrix CFX | Revision with same implant and open reduction |
| Luxation | Major | Biomedtrix Hybrid | Revision with same implant and open reduction |
| Luxation | Major | Helica | Revision with same implant and open reduction |
| Luxation | Major | Kyon | Revision with same implant and open reduction |
| Luxation | Major | Biomedtrix Hybrid | Revision with same implant and open reduction |
| Luxation | Major | Biomedtrix CFX | Revision with same implant and open reduction |
| Luxation | Major | Biomedtrix Hybrid | Revision with same implant and open reduction for explantation |
| Luxation | Major | Kyon | Open reduction and revision with new Kyon head-neck extra-long size |
| Luxation | Major | Kyon | Closed reduction |
| Luxation | Major | Kyon | Closed reduction |
| Luxation | Major | Biomedtrix CFX | Open reduction |
| Luxation | Major | Biomedtrix CFX | Explantation for open reduction (also had prosthesis related sepsis) |
| Neuropathic pain | Minor | Biomedtrix BFX | No action reported |
| Osteoarcoma found in distal left femur and Osteolysis of lateral cortex and medulla | Major | Biomedtrix CFX | Explantation and biopsy. |
| Poor function of prosthesis | Major | Biomedtrix BFX | Explantation |
| possible femoral fissure | Major | Biomedtrix BFX | Fissure fracture repair (Cerclage) |
| Prosthesis breakage | Major | Kyon | Explantation and revision with new implant (also had prosthesis breakage) |
| Prosthesis breakage | Major |  | Revision with new implant (also had acetabular cup displacement) |
| Prosthesis displaced | Major | Biomedtrix Hybrid | N/A |
| Prosthesis related sepsis | Major | Biomedtrix CFX | Explantation for open reduction (also had luxation) |
| Prosthesis related sepsis | Major | Biomedtrix BFX | Explantation |
| Prosthesis related sepsis | Major | Biomedtrix CFX | Explantation |
| Prosthesis related sepsis | Major | Biomedtrix CFX | Explantation |
| Prosthesis related sepsis | Major | Helica | Explantation |
| Prosthesis related sepsis | Major | Kyon | Explantation |
| Prosthesis related sepsis | Major | Kyon | Explantation and revision with new implant (also had sepsis) |
| Prosthesis related sepsis | Major | Helica | Revision with same implant and explantation (also had aseptic loosening) |
| Prosthesis related sepsis | Major | Biomedtrix BFX | Antibiotics |
| Prosthesis related sepsis | Major | Biomedtrix BFX | Explantation |
| Prosthesis related sepsis | Major | Kyon | Open lavage with gentamicin sponge placement and explantation |
| Prosthesis related sepsis | Major | Kyon | Revision with new implant. Replaced With a new acetabular cup with separate shell and liner. Replaced also with a new head size (also had aseptic loosening) |
| Prosthesis related sepsis | Major | Biomedtrix BFX | Revision with new implant and open reduction for explantation (also had luxation) |
| Prosthesis related sepsis | Major | Biomedtrix Hybrid | Revision with new implant (n=2) and open reduction for explantation (also had luxation) |
| Prosthesis related sepsis | Major |  | Revision with new implant |
| Recurrent luxation | Major | Kyon | Closed reduction followed by explantation for open reduction, and change H&N for closed reduction |
| Recurrent luxation | Major | Biomedtrix Hybrid | Revision with new implant and open reduction for explantation |
| Recurrent luxation | Major | Biomedtrix CFX | Revision with new implant (n=2) and open reduction for explantation |
| Recurrent luxation | Major |  | Explantation for open reduction and fracture at surgery repaired |
| Recurrent luxation | Major | Biomedtrix CFX | Open reduction for explantation |
| Recurrent luxation | Major | Biomedtrix BFX | Open reduction, revision with same implant and new implant |
| Recurrent luxation | Major | Biomedtrix BFX | Open reduction, revision with same implant and new implant |
| Sciatic neurapraxia | Minor | Biomedtrix CFX | No action reported |
| Sciatic neurapraxia | Minor | Biomedtrix CFX | No action reported |
| Sciatic neurapraxia | Major | Biomedtrix BFX | N/A |
| Sciatic neuropraxia | Minor | Biomedtrix CFX | Conservative treatment |
| sciatic neuropraxia | Minor | Kyon | No action reported |
| sciatic neuropraxia | Minor | Biomedtrix CFX | No action reported |
| Sciatic neuropraxia | Minor | Biomedtrix Hybrid | Conservative treatment |
| Sciatic neuropraxia | Minor | Biomedtrix CFX | Not reported |
| Sciatic neuropraxia | Minor |  | Not reported |
| Sciatic neuropraxia | Minor | Biomedtrix CFX | Conservative treatment. |
| Sciatic neuropraxia | Minor | Biomedtrix Hybrid | Fissure fracture repair (Cerclage) |
| Sciatic neuropraxia | Minor | Biomedtrix BFX | Conservative treatment |
| Sciatic neuropraxia | Minor | Biomedtrix CFX | Revision with new implant and open reduction for explantation |
| Sciatic neuropraxia | Major | Biomedtrix BFX | No action reported |
| Synoviocoele | Minor | Biomedtrix Hybrid | No action reported |
| Wound dehiscence | Major | Biomedtrix CFX | wound repair |
| Wound related sepsis | Major | Biomedtrix Hybrid | Antibiotics |
