# Supplementary material for: Outcomes and complications reported from a multiuser canine hip replacement registry over a 10‐year period
Source: Vet Surg. 2022 Sep 5;52(2):196–208. doi: 10.1111/vsu.13885 (PMC10087566; doi:10.1111/vsu.13885)
Supplement: Supplementary file 6 — Table S6 [file VSU-52-196-s001.docx]

| Breed | Number | Percentage |
| --- | --- | --- |
| Airedale Terrier | 2 | 0.4 |
| Akita | 3 | 0.7 |
| American Staffordshire Terrier | 1 | 0.2 |
| Anatolian Shepherd Dog | 1 | 0.2 |
| Argentinian Mastiff | 1 | 0.2 |
| Basenji | 1 | 0.2 |
| Bearded Collie | 1 | 0.2 |
| Belgian Malinois | 1 | 0.2 |
| Bernese Mountain Dog | 8 | 1.7 |
| Bichon Frise | 2 | 0.4 |
| Border Collie | 44 | 9.5 |
| Border Terrier | 1 | 0.2 |
| Boxer | 4 | 0.9 |
| Bulldog | 1 | 0.2 |
| Cairn Terrier | 1 | 0.2 |
| Cane Corso | 6 | 1.3 |
| Canine- Giant Poodle | 1 | 0.2 |
| Cavalier King Charles Spaniel | 4 | 0.9 |
| Czechoslovakian Wolfdog | 1 | 0.2 |
| Central Asian Shepherd | 1 | 0.2 |
| Chow Chow | 4 | 0.9 |
| Cockapoo | 1 | 0.2 |
| Cocker Spaniel | 9 | 2.0 |
| Cockerpoo | 3 | 0.7 |
| Crossbreed | 77 | 16.7 |
| Dogue De Bordeaux | 1 | 0.2 |
| English Cocker Spaniel | 4 | 0.9 |
| English Setter | 3 | 0.7 |
| English Springer Spaniel | 5 | 1.1 |
| Flat Coated Retriever | 1 | 0.2 |
| Fox Terrier | 1 | 0.2 |
| German Shepherd Dog | 56 | 12.1 |
| Giant Schnauzer | 1 | 0.2 |
| Golden Retriever | 30 | 6.5 |
| Gordon Setter | 1 | 0.2 |
| Irish Setter | 3 | 0.7 |
| Jack Russell Terrier | 3 | 0.7 |
| Kelpie | 1 | 0.2 |
| Labradoodle | 5 | 1.1 |
| Labrador Retriever | 7 | 1.5 |
| Labrador Retriever (Black) | 26 | 5.6 |
| Labrador Retriever (Chocolate) | 15 | 3.3 |
| Labrador Retriever (Yellow) | 38 | 8.2 |
| Lagotto Romagnolo | 2 | 0.4 |
| Lurcher | 3 | 0.7 |
| Maremmano Abruzzese | 4 | 0.9 |
| Miniature Poodle | 1 | 0.2 |
| Miniature Dachshund | 1 | 0.2 |
| Neapolitan Mastiff | 1 | 0.2 |
| Newfoundland | 3 | 0.7 |
| Northern Inuit | 1 | 0.2 |
| Old English Sheepdog | 3 | 0.7 |
| Other | 1 | 0.2 |
| Otterhound | 7 | 1.5 |
| Cane di Oropa | 1 | 0.2 |
| Patterdale Terrier | 1 | 0.2 |
| Pekingese | 1 | 0.2 |
| Pitbull | 1 | 0.2 |
| Pointer | 1 | 0.2 |
| Pomeranian | 1 | 0.2 |
| Portuguese Estrela | 1 | 0.2 |
| Pug | 1 | 0.2 |
| Red Setter | 1 | 0.2 |
| Rhodesian Ridgeback | 1 | 0.2 |
| Rottweiler | 10 | 2.2 |
| Samoyed | 1 | 0.2 |
| Siberian Husky | 2 | 0.4 |
| Spaniel | 1 | 0.2 |
| Springador | 1 | 0.2 |
| Springer Spaniel | 3 | 0.7 |
| Staffordshire Bull Terrier | 4 | 0.9 |
| Swiss Hound Dog | 1 | 0.2 |
| Swiss Shepherd Dog | 1 | 0.2 |
| Tibetan Terrier | 1 | 0.2 |
| Vizsla | 2 | 0.4 |
| Weimaraner | 3 | 0.7 |
| Welsh Springer Spaniel | 1 | 0.2 |
| West Highland White Terrier | 7 | 1.5 |
| Yorkshire Terrier | 4 | 0.9 |
| Not specified | 2 | 0.4 |
| Subtotal | 461 | 100 |
