# Supplementary material for: Outcomes and complications reported from a multiuser canine hip replacement registry over a 10‐year period
Source: Vet Surg. 2022 Sep 5;52(2):196–208. doi: 10.1111/vsu.13885 (PMC10087566; doi:10.1111/vsu.13885)
Supplement: Supplementary file 7 — Table S7 [file VSU-52-196-s003.docx]

| Disease | Frequency |
| --- | --- |
| Liver disease and atypical Cushing’s | 1 |
| Food intolerances | 1 |
| Allergies | 3 |
| Anal furunculosis | 1 |
| Problem with anal glands | 1 |
| Arthritis and skin allergy | 1 |
| Asthma | 1 |
| Back problems | 1 |
| Breathing problems | 1 |
| Bulging of the lumbosacral disc | 3 |
| Cancer | 5 |
| Canine discoid lupus | 1 |
| Contracted gracilis muscle | 1 |
| Crystalluria (calcium oxalate) | 1 |
| Epilepsy | 5 |
| Elbow problems and inflammatory bowel disease | 1 |
| Enlarged prostate | 1 |
| Heart murmur | 2 |
| Irritable bowel syndrome | 1 |
| Immune and digestive disease | 2 |
| Blindness and deafness | 1 |
| Pancreatitis | 1 |
| Pannus | 1 |
| Spinal problems | 1 |
| Spondylosis | 3 |
| Syringomyelia and dry eye | 1 |
| Torn tendon in shoulder | 1 |
| Twisted femur, patella laxation, and elbow thickening | 1 |
| Under active thyroid and polyarthritis | 1 |
| Vulvoplasty | 1 |
| Water filled cyst affecting spinal nerves | 1 |
| Autoimmune arthritis | 1 |
| Cherry eye | 1 |
| Chronic colitis, food intolerances, and allergic reactions | 1 |
| Dermatitis | 1 |
| Hernias | 1 |
| Gastroenteritis | 2 |
| Parasite infection such as giardia and mange. It also had gastroenteritis | 1 |
| Leishmania | 1 |
| Spinal meningitis | 1 |
| Recurrent otitis | 1 |
| Parvovirus | 1 |
| Uterine cancer and gastric torsion | 1 |
| Arthritis | 12 |
| Cruciate ligament disease | 5 |
| Elbow dysplasia | 7 |
| Elbow dysplasia and bilateral cruciate disease | 1 |
| Fracture | 2 |
| Had knee surgery | 1 |
| Patella luxation | 1 |
| Twisted femur, patella laxation, and elbow bone thickening | 1 |
| Genetic microfractures of the shoulders (not symptomatic) | 1 |
| Problems with the elbows | 1 |
