# Supplementary material for: Outcomes and complications reported from a multiuser canine hip replacement registry over a 10‐year period
Source: Vet Surg. 2022 Sep 5;52(2):196–208. doi: 10.1111/vsu.13885 (PMC10087566; doi:10.1111/vsu.13885)
Supplement: Supplementary file 8 — Table S8 [file VSU-52-196-s008.docx]

| Complication | Implant system | Treatment | Minor or major | Number of cases |
| --- | --- | --- | --- | --- |
| Fracture of the femur | Kyon | Treatment of complication was successful | Minor | 1 |
| Fracture of the femur | BioMedtrix Hybrid | Complication required further surgery | Major | 2 |
| Fracture of the femur | Kyon | Complication required further surgery | Major | 1 |
| Fracture of the femur | BioMedtrix BFX | Treatment of complication was successful | Major | 2 |
| Fracture of the femur | BioMedtrix BFX | Treated by a specialist veterinarian | Major | 1 |
| Fracture of the femur | BioMedtrix CFX | Complication required further surgery | Major | 1 |
| Fracture of the femur | BioMedtrix CFX | Treated by a local vet | Major | 1 |
| Infection of the prosthesis | Kyon | Treated by a specialist veterinarian | Minor | 3 |
| Infection of the prosthesis | Kyon | Treated by a specialist veterinarian and complication required further surgery | Major | 2 |
| Infection of the prosthesis | BioMedtrix CFX | Treatment of complication was successful | Minor | 1 |
| Infection of the prosthesis | BioMedtrix CFX | Explantation of the implant and complication required further surgery | Major | 1 |
| Infection of the prosthesis | BioMedtrix CFX | Treated by a local veterinarian | Minor | 1 |
| Loosening of the artificial hip (beyond 6 months after surgery) | BioMedtrix CFX | Complication required further surgery | Major | 1 |
| Loosening of the artificial hip (beyond 6 months after surgery) | BioMedtrix CFX |  | Minor | 1 |
| Loosening of the artificial hip (beyond 6 months after surgery) | Kyon | Complication required further surgery | Major | 1 |
| Loosening of the artificial hip (beyond 6 months after surgery) | BioMedtrix CFX | Explantation of the implant | Major | 2 |
| Loosening of the artificial hip (beyond 6 months after surgery) | BioMedtrix BFX | Treated by a specialist veterinarian | Major | `1 |
| Loosening of the artificial hip (beyond 6 months after surgery) | BioMedtrix BFX | Explantation of the implant and complication required further surgery | Major | 1 |
| Loosening of the artificial hip (within 3 months of surgery) | BioMedtrix CFX | Explantation of the implant | Major | 1 |
| Loosening of the artificial hip (within 3 months of surgery) | Kyon | Treatment of complication was successful | Major | 1 |
| Loosening of the artificial hip (within 3 months of surgery) | BioMedtrix BFX | Explantation of the implant | Major | 1 |
| Loosening of the artificial hip (within 3 months of surgery) | Helica | Complication required further surgery | Major | 2 |
| Loosening of the artificial hip (within 3 months of surgery) | BioMedtrix BFX | Treatment of complication was successful | Minor | 1 |
| Loosening of the artificial hip (within 3 months of surgery) and Infection of the artificial hip | BioMedtrix CFX | Explantation of the implant and complication required further surgery | Major | 1 |
| Loosening of the artificial hip (within 3 months of surgery) and Infection of the artificial hip | Helica | Explantation of the implant | Major | 1 |
| Loosening of the artificial hip (within 3 months of surgery) and luxation of the prosthesis | Kyon | Explantation of the implant and complication required further surgery. | Major | 1 |
| Loosening of the artificial hip (within 3 months of surgery), Luxation and Fracture of the femur | BioMedtrix CFX | Explantation of the implant, treated by a specialist veterinarian | Major | 1 |
| Loosening of the artificial hip (within 6 months of surgery and infection of the prothesis | BioMedtrix CFX | Treated by a local veterinarian and complication required further surgery | Major | 1 |
| Loosening of the artificial hip (within 6 months of surgery), Luxation and Fracture of the femur | BioMedtrix Hybrid | Treated by a local veterinarian and complication required further surgery | Major | 1 |
| Luxation and Loosening of the artificial hip (beyond 6 months after surgery) | Helica | Explantation of the implant and complication required further surgery | Major | 2 |
| Luxation of the prosthesis | BioMedtrix CFX | Complication required further surgery | Major | 7 |
| Luxation of the prosthesis | BioMedtrix CFX | Treated by a specialist veterinarian | Major | 1 |
| Luxation of the prosthesis | BioMedtrix CFX | Revision with the same implant | Major | 1 |
| Luxation of the prosthesis | Kyon | Complication required further surgery | Major | 3 |
| Luxation of the prosthesis | Kyon | Explantation of the implant and complication required further surgery | Major | 1 |
| Luxation of the prosthesis | BioMedtrix Hybrid | Complication required further surgery | Major | 2 |
| Luxation of the prosthesis | BioMedtrix BFX | Treated by a specialist veterinarian | Major | 3 |
| Luxation of the prosthesis | BioMedtrix BFX | Complication required further surgery | Major | 2 |
| Luxation of the prosthesis | BioMedtrix BFX | Treated by a specialist veterinarian | Minor | 1 |
| Luxation of the prosthesis | Kyon | Treated by a specialist veterinarian | Minor | 1 |
| Luxation of the prosthesis and fracture of femur | BioMedtrix BFX | Treated by a specialist veterinarian and complication required further surgery | Major | 1 |
| Other | BioMedtrix Hybrid | Complication required further surgery | Major | 1 |
| Other | BioMedtrix  BFX | Complication required further surgery | Major | 2 |
| Other | Kyon | Treated by a local veterinarian | Minor | 1 |
| Other | Kyon | Explantation of the implant | Major | 1 |
| Other | Kyon | Treatment of complication was successful | Minor | 2 |
| Other | BioMedtrix CFX | Complication required further surgery | Major | 2 |
| Other | Kyon | Treated by a specialist veterinarian | Minor | 2 |
| Other | Kyon | Complication required further surgery | Major | 2 |
| Other | Helica | Explantation of the implant | Major | 1 |
| Problems with skin wound healing | Kyon | Treated by a specialist veterinarian | Minor | 4 |
| Problems with skin wound healing | Helica | Treated by a specialist veterinarian | Minor | 1 |
| Problems with skin wound healing | Kyon | Treated by a local veterinarian | Minor | 2 |
| Problems with skin wound healing | Kyon | Treatment of complication was successful | Minor | 1 |
| Problems with skin wound healing | Kyon | Treatment of complication was successful | Major | 1 |
| Problems with skin wound healing | BioMedtrix CFX | Treated by a specialist veterinarian | Minor | 1 |
| Problems with skin wound healing | BioMedtrix CFX | Treated by a local veterinarian | Minor | 2 |
| Problems with skin wound healing and Loosening of the artificial hip (within 6 months of surgery). | BioMedtrix BFX | Complication required further surgery | Major | 1 |
| Problems with skin wound healing and Luxation of the prosthesis | Kyon | Treated by a specialist veterinarian | Minor | 1 |
| Problems with skin wound healing, infection and Luxation of the prosthesis | BioMedtrix CFX | Explantation of the implant and treatment required further surgery | Major | 1 |
| Problems with skin wound healing, Loosening of the artificial hip (beyond 6 months after surgery) | BioMedtrix CFX | Explantation of the implant | Major | 1 |
| Problems with skin wound healing, Loosening of the artificial hip (within 3 months of surgery) and Luxation of the prosthesis | Kyon | Explantation of the implant | Major | 1 |
| Problems with skin wound healing, Loosening of the artificial hip (within 3 months of surgery) and Luxation of the prosthesis | BioMedtrix Hybrid | Explantation of the implant | Major | 1 |
| Problems with skin wound healing, Loosening of the artificial hip (within 3 months of surgery), Infection and Luxation of the prosthesis | BioMedtrix  Hybrid | Explantation of the imlpant and treatment required further surgery | Major | 1 |
